# A Sulfuryl Group Transfer Strategy to Selectively Prepare Sulfated Steroids and Isotopically Labelled Derivatives

**DOI:** 10.3389/fmolb.2021.776900

**Published:** 2021-12-24

**Authors:** Jaber A. Alshehri, Daniel M. Gill, Alan M. Jones

**Affiliations:** Molecular Synthesis Laboratory, School of Pharmacy, Institute of Clinical Sciences, University of Birmingham, Edgbaston, United Kingdom

**Keywords:** sulfation, selectivity, isotopic labelling, sulfuryl transfer, TBSAB

## Abstract

The treatment of common steroids: estrone, estradiol, cortisol, and pregnenolone with tributylsulfoammonium betaine (TBSAB) provides a convenient chemoselective conversion of the steroids alcohol/phenol moiety to the corresponding steroidal organosulfate. An important feature of the disclosed methodology is the millimolar scale of the reaction, and the isolation of the corresponding steroid sulfates as their biologically relevant sodium salts without the need for ion-exchange chromatography. The scope of the method was further explored in the estradiol and pregnanediol steroid systems with the bis-sulfated derivatives. Ultimately, a method to install an isotopic label, deuterium (^2^H) combined with estrone sulfation is a valuable tool for its mass-spectrometric quantification in biological studies.

## Introduction

The preparation of authentic reference samples of sulfated steroids with either regioselective mono or di-sulfation patterns, (Lightning et al., 2021) combined with methods to isotopically label the resulting sulfated steroids is an ongoing challenge to their biological study. The resulting authentic sulfated steroids are key reference standards of paramount importance to the understanding of sulfatases (Mueller et al., 2015), (Günal et al., 2019), (Foster and Mueller, 2018), the role of steroid sulfation in diseases (Mueller et al., 2021) and the fields of detection of steroids, whether in abuse (Waller and McLeod, 2014) or in the environment, (Petrie et al., 2013) using spectroscopic techniques (Hill et al., 2019). Furthermore, the development of improved sulfation methods can be applied to both sulfated steroid containing natural products synthesis and structural elucidation studies (Hoye et al., 2007).

**CHART 1:**
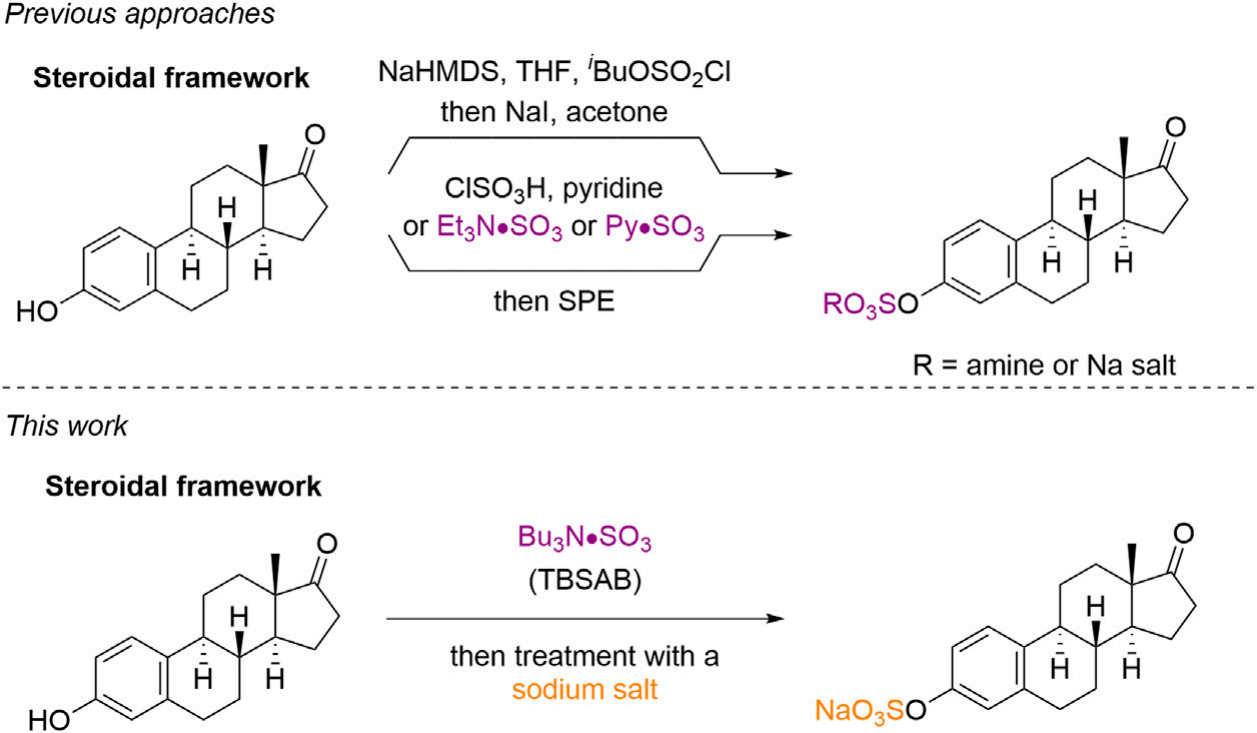
Current approaches to steroid sulfation and this work using TBSAB.

Current methods to sulfate steroids fall into two main categories (Chart 1). The use of a protected sulfate group (e.g., *iso*butyl protected sulfate esters) with subsequent deprotection (Simpson and Widlanski, 2006), or the use of a sulfur trioxide equivalent (e.g., chlorosulfonic acid or pyridine-sulfur trioxide complex) (Waller and McLeod, 2014), (Hungerford et al., 2006). Although these methods are effective, they suffer from the additional steps of deprotection and/or purification cascades. Issues with toxicity regarding pyridine contamination from the use of pyridine-sulfur trioxide complex in related carbohydrate scaffolds (Gabriel et al., 2020), (Vo et al., 2021) requires either an exceptionally vigilant isolation and analysis; or an improved overall method for steroid sulfation.

Our own current interest in the sulfation field derives from the development of tributylsulfoammonium betaine (TBSAB) (Gill et al., 2019a), (Jones, 2021) as a convenient one-pot method for the sulfation of heteroatom containing bioactive molecules. (Benedetti et al., 2020), (Alshehri et al., 2020) This was inititated due to challenges encountered with the purification of sulfated small molecule heparin sulfate glycomimetics (Gill et al., 2021), (Gill et al., 2019b), (Mahmoud et al., 2019), (Langford-Smith et al., 2019), (Mahmoud et al., 2017) with conventional, pre-existing sulfation methods. A key advantage of TBSAB over similar amine containing-sulfur trioxide complexes (e.g., triethylamine-sulfur trioxide) is the lipophilic nature of the counterion avoiding the need for ion-exchange chromatography. Herein we report our findings on the use of TBSAB as a general, scalable and regioselective sulfating reagent for steroids, and the application of TBSAB in conjugation with isotopic labelling for steroidal-organosulfate reference standards.

## Results and Discussion

**SCHEME 1:**
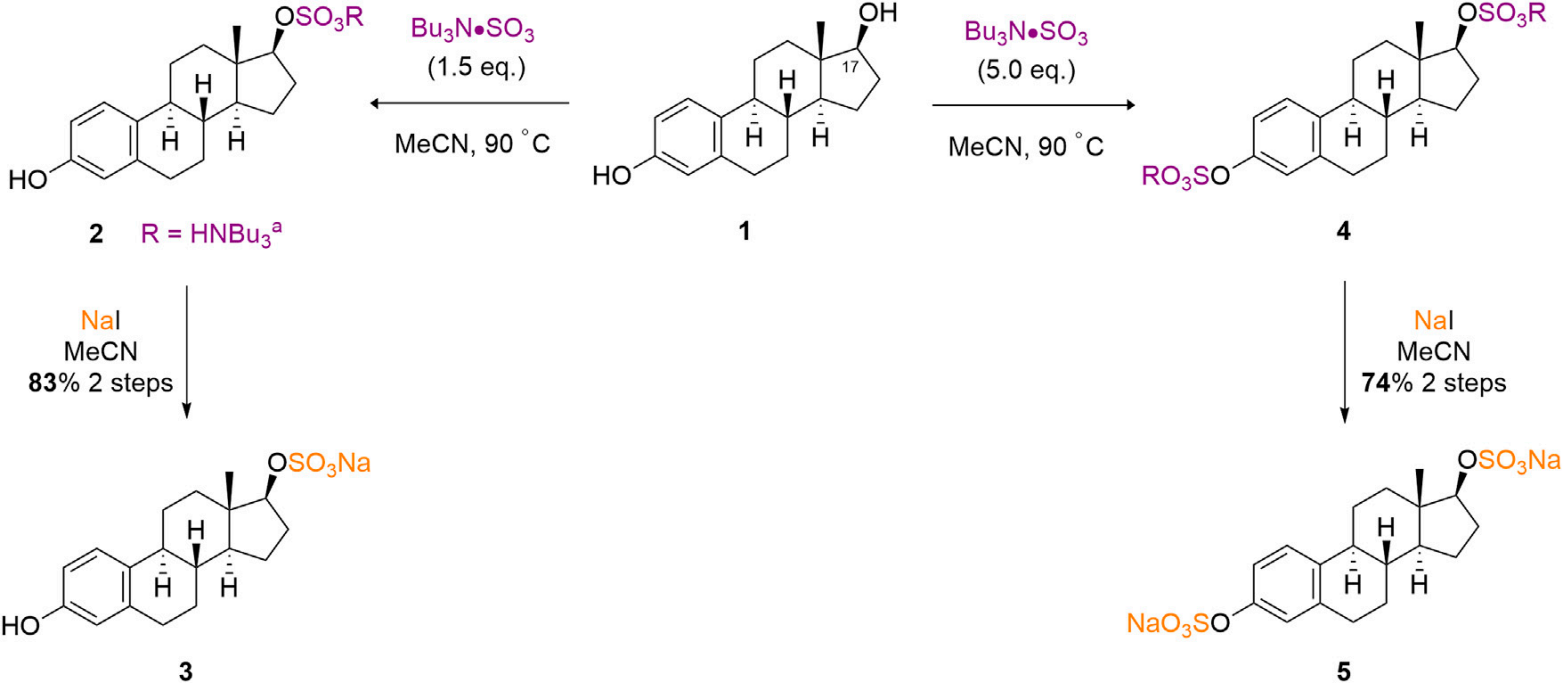
TBSAB mediated regioselective sulfation of β-estradiol **(1)** affords the mono- or double sulfated estradiols as their sodium salts.

Our initial exploration of the method builds upon early screening results of TBSAB, including a single example on β-estradiol (**1**) (Gill et al., 2019a). We firstly sought to demonstrate the reproducibility of this method on a 1.0 mmol scale, thus taking commercially available β-estradiol (**1**) and treating it with TBSAB resulted in exclusive C (17), secondary alcohol, sulfation (**2**). Furthermore the same conditions using an excess of TBSAB resulted in both C (17) sulfation and C (3), phenol, sulfation of (**4**) presumably occurs via initial C (17) alcohol sulfation in a stepwise installation. In both cases, a work-up using sodium iodide isolated the mono (**3**) and double (**5**) sulfated steroids as their sodium salts, in good yields without the risk of pyridinium ion contamination (Scheme 1).

**SCHEME 2:**
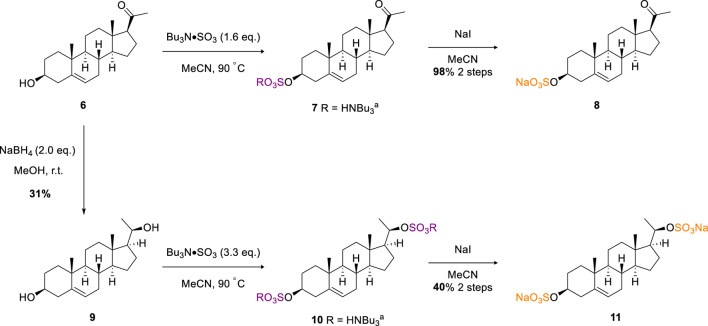
Sulfation of the pregnenolone **(6)** and pregnendiol **(9)** steroids.

Next, we considered sulfation of a more challenging biologically active substrate, pregnenolone (**6**). (Harteneck, 2013). Under analogous conditions to the β-estradiol examples, and on a 0.3 mmol scale, steroidal sulfate **8** was afforded after sodium exchange in an excellent 98% isolated yield (Scheme 2). Diastereoselective reduction of the ketone moiety of pregnenolone using sodium borohydride afforded pregnanediol in 31% yield (**9**). Crystallographic data of the bulk material from d_6_-DMSO crystallisation supports the assignment of the major diastereomer as *R* at the newly set stereocentre (Supplementary Figure S3) (2120263 contain, 2120). As **9** contains two secondary alcohol motifs, treatment with TBSAB afforded the double sulfated pregnanediol (**11**) in a modest 40% isolated yield on a 0.6 mmol scale.

**SCHEME 3:**
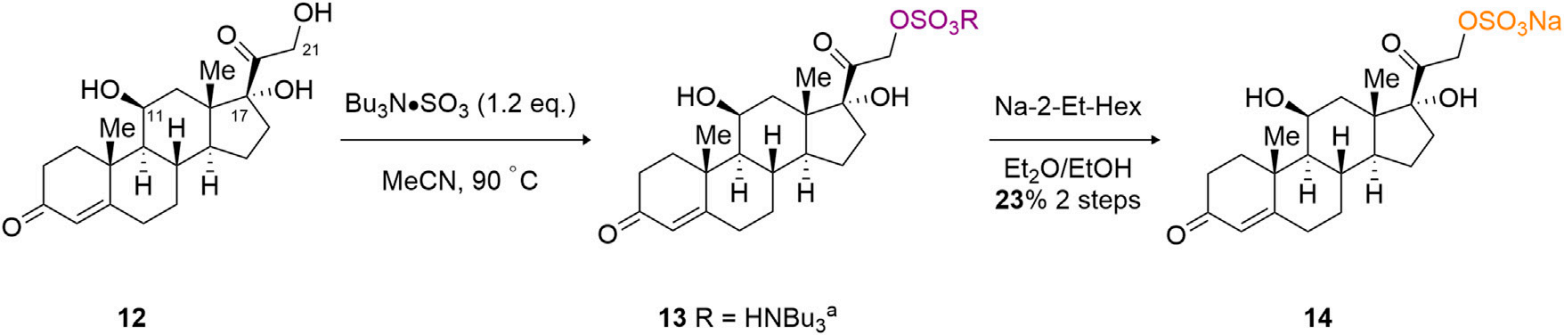
Regioselective C (21) sulfate ester formation on cortisol **(12)**.

The ultimate test of the TBSAB method, in relation to regioselective sulfation, is the complex triol, cortisol (**12**) (Scheme 3). Cortisol contains three potentially reactive hydroxyl motifs at the C (11), C (17) and C (21) positions. It was anticipated that a regioselective sulfation of the primary C (21) alcohol would result over the C (11), secondary, or C (17), tertiary, alcohol moieties, despite the presence of the α-ketone affecting the reactivity of the C (21)-OH. To our delight, a microscale (8 mg) treatment of cortisol with TBSAB afforded the C (21) organosulfate in a modest 17% overall yield (23% based on recovered starting material) as the sodium salt (**14**). Furthermore, no unwanted C (11) or indeed C (17) sulfate ester formation was observed.

**SCHEME 4:**
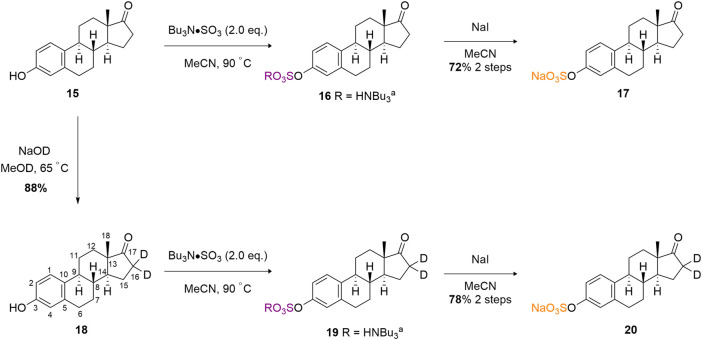
Estrone and estrone-d_2_ sulfation using TBSAB.

Finally, we sought to develop a proof-of-concept isotopic labelling-chemoselective sulfation method for the estrone scaffold (**15**) (Scheme 4). Prior to developing a deuterium labelling method at the C (16) methylene position, a model non-deuterated estrone was sulfated at the C (3) phenolic position in good 72% isolated yield as the sodium salt (**17**). A higher equivalence of TBSAB (2.0 eq) was used to ensure complete sulfation at the sole reactive C (3) phenolic centre. This provided confidence that sulfation should occur readily at the C (3) position using TBSAB on the deuterium labeled substrate.

**FIGURE 1:**
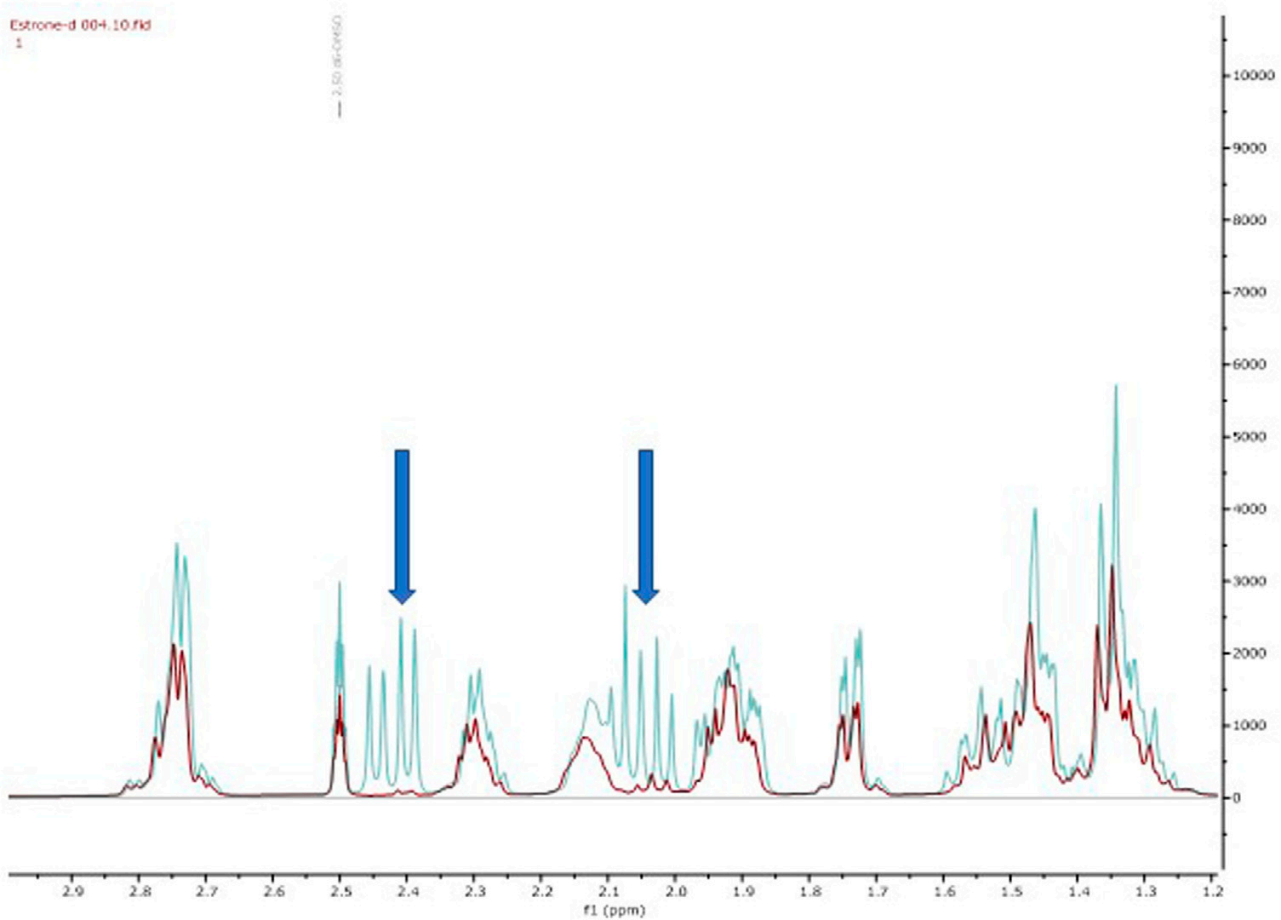
Overlay of ^1^H NMR spectra of estrone **(**blue, **15)** and estrone-d_2_ **(**red, **18)** shows the diagnostic reduction of the diastereotopic C (16) protons.

Firstly, we adapted the method of Rudqvist for C (19) deuteration (Rudqvist, 1983). Treatment of the estrone with NaOD in MeOD resulted in estrone-d_2_ formation (**18**). The C (16)-H_2_ protons were selectively deuterated by enolate formation with sodium deutroxide and resultant deuterium incorporation by quenching the enolate with methanol-D (CH_3_OD). This was confirmed via comparative 2D-NMR spectroscopic studies (see Supporting Information) but the key disappearance of the C (16) protons can be clearly observed in the ^1^H-NMR spectral overlay (Figure 1). It should be noted that deuteration next to a carbonyl group is not usually recommended for applied quantification studies as the deuterium label could readily back exchange through a keto-enol tautomerisation leading to a loss of the label (Wudy, 1990). In our system we observed, a decline of deuterium label in solution based mass-spectrometry studies (Supplementary Figures S1, S2).

Finally, the treatment of estrone-d_2_ with TBSAB afforded the sulfated and isotopically labelled estrone-d_2_ sulfate in 78% isolated yield and 67% incorportation of the deuterium label (**20**).

## Conclusion

In summary, we have demonstrated a general method for the synthesis of mono- or di-sulfated steroidal skeletons of importance to the fields of biology and spectroscopmetric detection. We have showcased chemo-selective sulfation within a variety of complex structures, such as cortisol, and developed a simplified deuterium labeling-sulfation strategy for estrone. Overall, these approaches provide tractable routes on preparative scales to multiple sulfated steroid classes as reference compounds for detection of substances of abuse through to cancer diagnosis applications.

## Data Availability

The original contributions presented in the study are included in the article/Supplementary Materials, further inquiries can be directed to the corresponding author fid files can be found at the following doi: https://doi.org/10.25500/edata.bham.00000720.
