# Corrigendum: A sulfuryl group transfer strategy to selectively prepare sulfated steroids and isotopically labelled derivatives

**DOI:** 10.3389/fmolb.2024.1504226

**Published:** 2024-12-19

**Authors:** Jaber A. Alshehri, Daniel M. Gill, Alan M. Jones

**Affiliations:** Molecular Synthesis Laboratory, School of Pharmacy, Institute of Clinical Sciences, University of Birmingham, Edgbaston, United Kingdom

**Keywords:** sulfation, selectivity, isotopic labelling, sulfuryl transfer, TBSAB

In the published article, there was an error in Scheme 2 as published. The stereogenic center in compounds 9-11 was incorrectly depicted. The corrected Scheme 2 and its caption appear below.

**SCHEME 2:**
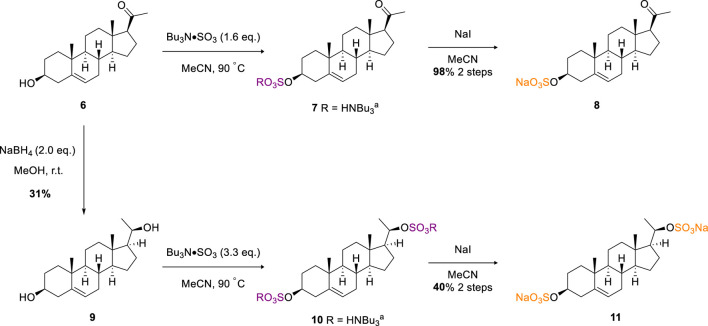
Sulfation of the pregnenolone (6) and pregnendiol (9) steroids.

In the published article, there was an error in **Supplementary Data Sheet 1**, section Compound characterisation. The name of the fourth compound was incorrectly captured as “Pregnanediol or (3S,8S,9S,10R,13S,14S,17S)-17-((S)-1-hydroxyethyl)-10,13-dimethyl-2,3,4,7,8,9,10,11,12,13,14,15,16,17-tetradecahydro-1H-cyclopenta[a]phenanthren-3-ol (**9**).”

The correct name appears below:

“Pregnanediol or (3*S*,8*S*,9*S*,10*R*,13S,14*S*,17*S*)-17-((*R*)-1-hydroxyethyl)-10,13-dimethyl-2,3,4,7,8,9,10,11,12,13,14,15,16,17-tetradecahydro-1*H*-cyclopenta[*a*]phenanthren-3-ol (**9**).”

In the published article, there was an error in **Supplementary Data Sheet 1**, section Compound characterisation. The name of the fifth compound was incorrectly captured as “Disodium-3,17-pregnanediol disulfate or sodium (*S*)-1-((3*S*,8*S*,9*S*,10*R*,13*S*,14*S*,17*S*)-10,13-dimethyl-3-(sulfonatooxy)-2,3,4,7,8,9,10,11,12,13,14,15,16,17-tetradecahydro-1H-cyclopenta[*a*]phenanthren-17-yl)ethyl sulfate (**11**).”

The correct name appears below:

“Disodium-3,17-pregnanediol disulfate or sodium (3*S*,8*S*,9*S*,10*R*,13*S*,14*S*,17*S*)-10,13-dimethyl-17-((*R*)-1-(sulfonatooxy)ethyl)-2,3,4,7,8,9,10,11,12,13,14,15,16,17-tetradecahydro-1*H*-cyclopenta[*a*]phenanthren-3-yl sulfate (**11**).”

The authors apologize for these errors and state that these do not change the scientific conclusions of the article in any way. The original article has been updated.

