# Supplementary material for: A Sulfuryl Group Transfer Strategy to Selectively Prepare Sulfated Steroids and Isotopically Labelled Derivatives
Source: Front Mol Biosci. 2021 Dec 24;8:776900. doi: 10.3389/fmolb.2021.776900 (PMC8740147; doi:10.3389/fmolb.2021.776900)
Supplement: Supplementary file 1 [file DataSheet1.pdf]

Supplementary Material

A sulfuryl group transfer strategy to selectively prepare sulfated steroids and isotopically labelled derivatives

Jaber A. Alshehri[1], Daniel M. Gill[1], Alan M. Jones[1*]

[1]Molecular Synthesis Laboratory, Institute of Clinical Sciences, School of Pharmacy, University of Birmingham, Edgbaston, United Kingdom

*** Correspondence:**
Dr Alan M. Jones
a.m.jones.2@bham.ac.uk

CONTENTS

General methods	page 2
General procedures	page 2-3
Compound characterisation	page 3-10
Copies of NMR spectra	pages 11-38
Supplementary Figures	pages 39-41
X-ray crystallographic data for compound **9**	pages 42-43
References	page 44

General Methods

All reactions involving moisture sensitive reagents were carried out using standard Schlenk techniques, in a dry reaction vessel under argon. All solvents used under anhydrous conditions were decanted directly from an SPS dispensary or were stored over 4 Å molecular sieves 24 h prior to use.

Solvents used for workup procedures were of technical grade from Sigma-Aldrich, Honeywell, VWR or Fisher Scientific. Unless stated otherwise, solvents were removed by rotary evaporation under reduced pressure between 30-50 °C. All chemical reagents were used as received unless stated otherwise. Reactions were monitored by TLC analysis on Merck silica gel 60 F254 using UV light (254 nm) and/or potassium permanganate.

^{1}H and ^{13}C NMR spectra were recorded either on a Bruker AVIII operating at 300 MHz for ^{1}H and fitted with a 5mm BBFO probe or on a Bruker AVANCE NEO operating at 400 MHz for ^{1}H fitted with a 5mm "smart" BBFO probe, respectively. ^{1}H-^{1}H COSY, DEPT-45, ^{1}H-^{13}C HSQC, and ^{1}H-^{13}C HMBC NMR spectra were recorded on a Bruker AVANCE NEO console operating at 400 MHz for 1H and fitted with a nitrogen-cooled BBFO probe. Chemical shift data for ^{1}H are reported in parts per million (ppm, δ scale) downfield from tetramethylsilane (TMS: δ 0.0) and referenced internally to the residual proton in the solvent. The deuterated solvents used for NMR analysis were chloroform ($CDCl_3$:δH 7.26, δC 77.2) and dimethyl sulfoxide (DMSO-d_6: δH 2.50, δC 39.5). Coupling constants (*J*) are given in hertz (Hz). The data are presented as follows: chemical shift, multiplicity (s = singlet, d = doublet, t = triplet, q = quartet, p = pentet, m = multiple, br = broad, app = apparent and combinations thereof), coupling constant and integration and assignment.

Mass spectra were recorded on a Waters Xevo G2-XS Tof or Synap G2-S mass spectrometer using Zspray, Electro-spray ionization in negative (ESI^-) and positive (ESI^+) mode, respectively.

General procedure 1. Synthetic procedure for preparation of sodium mono-sulfated steroids using Tributylsulfoammonium betaine (TBSAB).

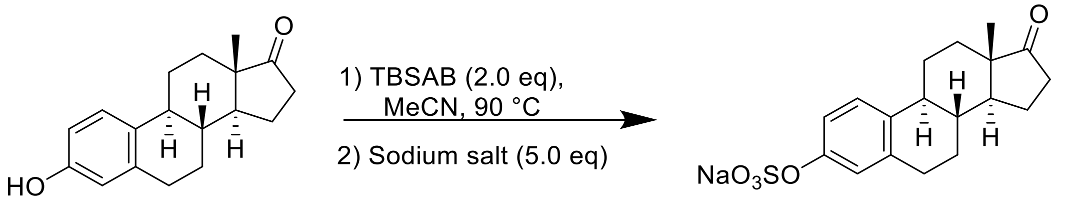

A flame dried 100 mL round bottom flask was charged with the appropriate steroid (1.0 mmol) and TBSAB (1.2-5.0 eq.) under argon. Anhydrous MeCN (1.5-4.0 mL per mmol of TBSAB) was added to the flask and the reaction mixture was heated under reflux until the TLC indicted that the reaction mixture was complete (typically 2-7 h). Then, the flask was cooled to room temperature and the solvent removed under reduced pressure to afford the desired sulfated steroid as its tributylammonium salt.

Work-up procedure A: The flask containing the tributylammonium salt was charged with EtOH (30 mL) and sodium 2-ethylhexanoate (5.0 eq). The reaction mixture was stirred vigorously for 1h at room temperature. The precipitate was removed by filtration, washed with EtOH (3 × 20 mL) and dried to a constant weight to afford the desired di-sulfated steroid as its sodium salt.

Work-up procedure B: The flask containing the tributylammonium salt was charged with MeCN (25 mL) and sodium iodide (5.0 eq). The reaction mixture was stirred vigorously for 1h at room temperature. The precipitate was removed by filtration, washed with MeCN (3 × 20 mL) and dried to a constant weight to afford the desired di-sulfated steroid as its sodium salt.

Compound characterisation

Sodium 17β-estradiol sulfate or Sodium(8*R*,9*S*,13*S*,14*S*,17*S*)-3-hydroxy-13-methyl-7,8,9,11,12,13,14,15,16,17-decahydro-6*H*-cyclopenta[*a*]phenanthren-17-yl sulfate (**3**)[S1]

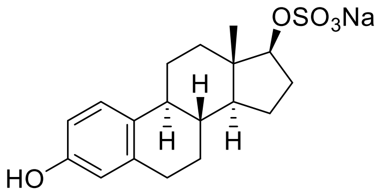

Following **general procedure 1:** β-Estradiol (**1**) (272.4 mg, 1.0 mmol) and TBSAB (398 mg, 1.5 mmol) were dissolved in anhydrous MeCN (4.0 mL) and heated under reflux for 3 hours. After the completion of reaction, the flask was cooled to room temperature and the solvent removed under reduced pressure. The contents were purified (SiO_2; CH_2Cl_2/MeOH, 9:1, R_f = 0.16) to yield an intermediate tributylammonium sulfate as a clear oil which then was purified by **work up procedure B** to yield the title compound as yellow powder (311 mg, 83%).

M.P. 157-159 °C (Lit. 170 °C)[S1]

^{1}H NMR (400 MHz, DMSO-d_6) δ 9.01 (s, 1H, O<u>H</u>), 7.04 (d, *J* = 8.5 Hz, 1H), 6.50 (dd, *J* = 8.5, 2.6 Hz, 1H), 6.42 (d, *J* = 2.6 Hz, 1H), 4.05 (t, *J* = 8.5 Hz, 1H), 2.80-2.63 (m, 2H), 2.29–2.13 (m, 1H), 2.16–1.83 (m, 3H), 1.84–1.70 (m, 1H), 1.66-1.44 (m, 2H), 1.33-1.06 (m, 6H), 0.68 (s, 3H).

^{13}C NMR (101 MHz, DMSO-d_6) δ 154.9, 137.1, 130.4, 126.1, 114.9, 112.7, 84.1, 49.2, 43.5, 42.4, 38.5, 36.6, 29.2, 28.2, 26.9, 26.0, 22.7, 11.7.

LRMS. *m/z* (ES-) 351.13 ([M-Na]$^-$, 100%), 352.13 ([M^{13}C-Na]$^-$, 20%).

HRMS. *m/z* (ES-) $C_{18}H_{23}O_5S$ requires 351.1276, found 351.1266 [M-Na]$^-$.

Data were in accordance with the literature.[S1]

Disodium-3,17β-estradiol disulfate or Sodium (8*R*,9*S*,13*S*,14*S*,17*S*)-13-methyl-7,8,9,11,12,13,14,15,16,17-decahydro-6*H*-cyclopenta[*a*]phenanthrene-3,17-diyl bis(sulfate)) (**5**)[S2]

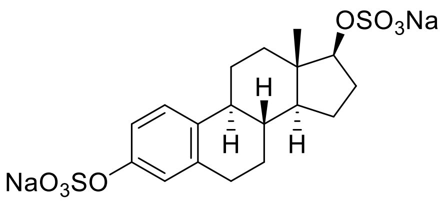

Following **general procedure 1:** β-Estradiol (**1**) (136.2 mg, 0.5 mmol) and TBSAB (663.5 mg, 2.5 mmol) were dissolved in anhydrous MeCN (4.0 mL) and heated under reflux until the TLC indicated that the reaction was complete. After the completion of reaction, the flask was cooled to room temperature and the solvent removed under reduced pressure. The crude product was purified by **work up procedure B** and recrystallized from MeOH/EtOAc afforded the title compound as a white solid (354 mg, 74%).

M.P. 162-164 °C (Lit. 137-140 °C)[S2]

^{1}H NMR (400 MHz, DMSO-d$_6$) δ δ 7.15 (d, *J* = 8.5 Hz, 1H), 6.93–6.75 (m, 2H), 4.07-4.03 (m, 1H), 2.85–2.67 (m, 2H), 2.31–2.17 (m, 1H), 2.17–2.09 (m, 1H), 2.09–1.87 (m, 2H), 1.87–1.70 (m, 1H), 1.69–1.48 (m, 2H), 1.44–1.05 (m, 6H), 0.70 (s, 3H).

^{13}C NMR (101 MHz, DMSO-d$_6$) δ 151.1, 136.1, 134.8, 125.5, 120.5, 118.0, 84.1, 49.2, 43.6, 42.4, 38.3, 36.6, 29.2, 28.2, 26.8, 25.9, 22.7, 11.7.

LRMS. *m/z* (ES$^-$) 215.06 ([M-2Na]$^{2-}$, 100%), 431.08 ([M -2Na+H]$^-$, 10%).

HRMS. *m/z* (ES$^-$) $C_{18}H_{23}O_8S_2$ requires 431.0848, found 431.0834 [M-2Na]$^{2-}$.

Data were in accordance with the literature.[S2]

Sodium-3-pregnenolone sulfate or Sodium (3*S*,8*S*,9*S*,10*R*,13*S*,14*S*,17*S*)-17-acetyl-10,13-dimethyl-2,3,4,7,8,9,10,11,12,13,14,15,16,17-tetradecahydro-1H-cyclopenta[*a*]phenanthren-3-yl sulfate (**8**)[S3]

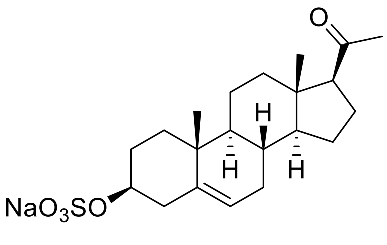

Following **general procedure 1:** Pregnenolone (**6**) (100 mg, 0.3 mmol) and TBSAB (132.7 mg, 0.5 mmol) were dissolved in anhydrous MeCN (2.0 mL) and heated under reflux until the TLC indicated that the reaction was complete. After the completion of reaction, the flask was cooled to room temperature and the solvent removed under reduced pressure. The crude product was purified by **work up procedure B** to yield the title compound as white solid (194 mg, 98%).

M.P. 121-123 °C

^{1}H NMR (400 MHz, DMSO-d$_6$) δ 5.29 (dt, *J* = 5.0, 2.1 Hz, 1H), 3.84 (tt, *J* = 11.5, 4.6 Hz, 1H), 2.57 (t, *J* = 8.9 Hz, 1H), 2.37 (ddd, *J* = 13.3, 4.9, 2.1 Hz, 1H), 2.19 – 2.10 (m, 1H), 2.08-2.04 (m, 4H), 2.04 –

1.97 (m, 2H), 1.97 – 1.84 (m, 2H), 1.81 (dt, J = 13.3, 3.5 Hz, 1H), 1.64 – 1.50 (m, 4H), 1.48 – 1.33 (m, 4H), 1.25 – 1.07 (m, 2H), 1.01 (dd, J = 13.8, 3.8 Hz, 1H), 0.94 (s, 3H), 0.53 (s, 3H).

^{13}C NMR (101 MHz, DMSO-d$_6$) δ 208.5, 140.7, 121.0, 75.2, 62.5, 56.0, 49.4, 43.3, 37.9 (2C), 36.9, 36.1, 31.3, 31.3, 31.2, 28.8, 24.0, 22.2, 20.6, 19.0, 12.9.

LRMS. *m/z* (ES$^-$) 395.19 ([M-Na]$^-$, 100 %), 396.19 ([M^{13}C-Na]$^-$, 20%).

HRMS. *m/z* (ES$^-$) $C_{21}H_{31}O_5S$ requires 395.1892, found 395.1907 [M-Na]$^-$.

Data were in accordance with the literature.[S3]

Pregnanediol or ((3*S*,8*S*,9*S*,10*R*,13S,14*S*,17*S*)-17-((*R*)-1-hydroxyethyl)-10,13-dimethyl-2,3,4,7,8,9,10,11,12,13,14,15,16,17-tetradecahydro-1*H*-cyclopenta[*a*]phenanthren-3-ol (**9**) [S4]

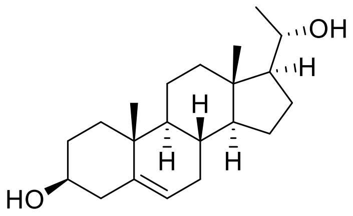

A round bottom flask was charged with pregnenolone (**6**) (1.0 g, 3.1 mmol) and MeOH (20 mL) were added to a flame- dried round bottom flask under inert environment. The sodium borohydride (240 mg, 6.3 mmol) was added in portions over 10 min and the reaction mixture was stirred for an additional 2 h. After the completion of reaction, the white precipitate was collected by filtration and dried for overnight to afford the desired product as a white solid (314 mg, 0.97 mmol, 31%)

M.P. 174-176 °C (Lit. 211-212 °C)[S4]

^{1}H NMR (300 MHz, DMSO-d$_6$) δ 5.25 (d, J = 4.8, 2.7 Hz, 1H), 4.59 (d, J = 3.9 Hz, 1H), 4.09 (d, J = 5.3 Hz, 1H), 3.54 – 3.41 (m, 1H), 3.30 – 3.19 (m, 1H), 2.19 – 2.05 (m, 3H), 1.95 – 1.84 (m, 1H), 1.81 – 1.71 (m, 1H), 1.71 – 1.62 (m, 1H), 1.60 – 1.48 (m, 3H), 1.47 – 1.31 (m, 5H), 1.27 – 1.16 (m, 1H), 1.15 – 1.03 (m, 3H), 0.99 (d, J = 6.1 Hz, 3H), 0.94 (s, 3H), 0.92 – 0.79 (m, 2H), 0.68 (s, 3H).

^{13}C NMR (101 MHz, DMSO-d$_6$) δ 141.3, 120.4, 70.0, 68.3, 57.6, 55.8, 49.8, 42.2, 41.8, 39.2, 36.9, 36.1, 31.5, 31.4, 31.4, 25.3, 24.2, 23.8, 20.4, 19.2, 11.9.

LRMS. *m/z* (TOF MS APAP$^+$) 283.25 ([M-2OH+H]$^+$, 100%), 284.25 ([M^{13}C-2OH+H]$^+$, 100%), 301.25 ([M-OH]$^+$, 70%), 302.26 ([M^{13}C -OH]$^+$, 20%).

HRMS. *m/z* (TOF MS APAP$^+$) $C_{21}H_{33}O$ requires 301.2531, found 301.2541 [M-OH]$^+$.

$[\propto]^{\mathbf{26}}_{\mathbf{589}}$ = -17.3 (c 1.23, 95% ethanol)

Data were in accordance with the literature.[S4]

Disodium-3,17-pregnanediol disulfate or sodium (3*S*,8*S*,9*S*,10*R*,13*S*,14*S*,17*S*)-10,13-dimethyl-17-((*R*)-1-(sulfonatooxy)ethyl)-2,3,4,7,8,9,10,11,12,13,14,15,16,17-tetradecahydro-1*H*-cyclopenta[*a*]phenanthren-3-yl sulfate (**11**)

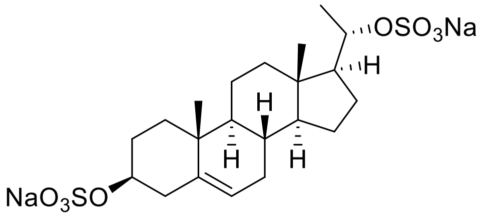

Following **general procedure 1:** Pregnanediol (**9**) (197 mg, 0.6 mmol) and TBSAB (530.8 mg, 2.0 mmol) were dissolved in anhydrous MeCN (4.0 mL) and heated under reflux until the TLC indicated that the reaction was complete. After the completion of reaction, the flask was cooled to room temperature and the solvent removed under reduced pressure. The crude product was purified by **work up procedure B** to yield the title compound as white solid (344 mg, 0.6 mmol, 40%).

M.P. 156-158 °C

^{1}H NMR (400 MHz, DMSO-d_6) δ 5.30 – 5.25 (m, 1H), 4.16 – 3.97 (m, 1H), 3.92–3.70 (m, 1H), 2.41–2.32 (m, 1H), 2.22 (d, *J* = 12.9 Hz, 1H), 2.18 – 2.04 (m, 1H), 1.95–1.76 (m, 3H), 1.62–1.49 (m, 3H), 1.49 – 1.26 (m, 6H), 1.20-1.15 (m, 1H), 1.13 (d, *J* = 6.0 Hz, 3H), 1.04 – 0.80 (m, 7H), 0.72 (s, 3H).

^{13}C NMR (101 MHz, DMSO-d_6) δ 140.8, 121.1, 75.3, 74.4, 56.1, 55.6, 49.7, 41.7, 38.0, 36.9, 36.1, 31.5, 31.4 (2C), 28.8, 25.0, 23.9, 20.5, 20.4, 19.0, 11.3.

LRMS. *m/z* (ES$^-$) 238.07 ([M-2Na]$^{2-}$, 100%), 239.08 ([M^{13}C-2Na]$^{2-}$, 100%), 477.16 ([M-2Na]$^-$, >1%), 478.16 ([M^{13}C -2Na+H]$^-$, <1%).

HRMS. *m/z* (ES$^-$) $C_{21}H_{32}O_8S_2$ requires 476.1550, found 476.1538 [M-2Na]$^-$.

Sodium-21-cortisol sulfate or Sodium 2-((8*S*,9*S*,10*R*,11*S*,13*S*,14*S*,17*R*)-11,17-dihydroxy-10,13-dimethyl-3-oxo-2,3,6,7,8,9,10,11,12,13,14,15,16,17-tetradecahydro-*1H*-cyclopenta[*a*]phenanthren-17-yl)-2-oxoethyl sulfate (**14**) [S5]

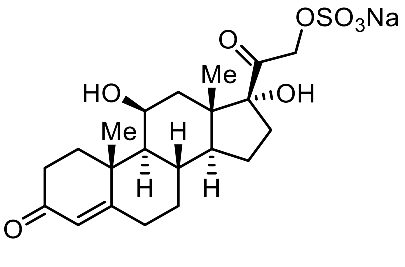

Cortisol (36 mg, 0.1 mmol) and TBSAB (32 mg, 0.13 mmol, 1.3 eq.) were dissolved in dry MeCN (0.2 mL) and heated under reflux for 2 h with monitoring by HPLC (C_{18} column, MeCN/H_2O 3:7). The flask was cooled and the solvent was removed under reduced pressure. The residue was purified

by chromatography (SiO_2, EtOAc/MeOH 19:1 to 2:1) to afford 24 mg of **38** and 12mg of cortisol. The intermediate (**38**) was purified by work up procedure **B** to afford the title compound as a white solid (8 mg, 17% (23% *b.r.s.m.*)).

^{1}H NMR (400 MHz, DMSO-d$_6$) δ 5.55 (d, *J* = 1.5 Hz, 1H), 5.31 (s, 1H), 4.85 (d, *J* = 18.2 Hz, 1H), 4.43 (d, *J* = 18.2 Hz, 1H), 4.37 (d, *J* = 3.2 Hz, 1H), 4.24 (d, *J* = 13.5 Hz, 1H), 2.43 – 2.35 (m, 2H), 2.23 – 2.12 (m, 2H), 2.11-2.04 (m, 1H), 1.95-1.86 (m, 3H), 1.83 – 1.73 (m, 1H), 1.71 – 1.59 (m, 2H), 1.59 – 1.51 (m, 1H), 1.42-1.32 (m, 4H), 1.31-1.21 (m, 2H), 0.90-0.83 (m, 2H), 0.75 (s, 3H).

^{13}C NMR (101 MHz, DMSO-d$_6$) δ 206.5, 198.3, 172.4, 121.5, 88.7, 69.7, 66.4, 55.5, 52.1, 47.3, 40.2, 35.7, 34.5, 33.5, 32.9, 32.8, 31.4, 31.2, 23.7, 21.0, 17.6.

LRMS *m/z* (ESI$^+$) 443.17 (100%, [M-Na+2H]$^+$).

HRMS *m/z* (ESI$^+$) $C_{21}H_{31}NaO_8S$ Requires: 443.1734, Found: 443.1746 ([M-Na+2H]$^+$).

Data were in accordance with the literature.[S5]

β-estrone (**15**)[S6]

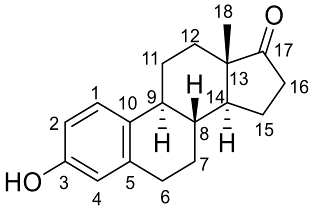

^{1}H NMR (400 MHz, DMSO-*d*$_6$) δ 9.03 (s, 1H, C3-O<u>H</u>), 7.07–7.01 (m, 1H, C1-<u>H</u>), 6.51 (dd, *J* = 8.4, 2.6 Hz, 1H, C2-<u>H</u>), 6.45 (d, *J* = 2.6 Hz, 1H, C4-<u>H</u>), 2.81-2.67 (m, 2H), 2.48–2.37 (m, 1H, C16-<u>H</u>), 2.34–2.25 (m, 1H, C11-<u>H</u>), 2.17–2.09 (m, 1H, C9-<u>H</u>), 2.04 (dd, *J* = 18.8, 8.9 Hz, 1H, C16-<u>H</u>), 1.98–1.92 (m, 1H, C15-<u>H</u>), 1.92–1.86 (m, 1H, C7-<u>H</u>), 1.77–1.67 (m, 1H, C12-<u>H</u>), 1.60–1.40 (m, 3H), 1.39–1.25 (m, 3H), 0.81 (s, 3H, C18-CH_3).

^{13}C NMR (101 MHz, DMSO-d$_6$) δ 219.7 (C17), 155.0 (C3), 137.1 (C5), 129.9 (C10), 126.0 (C1), 114.9 (C4), 112.8 (C2), 49.6 (C14), 47.3 (C13), 43.4 (C9), 37.9 (C8), 35.4 (C16), 31.3 (C12), 29.0 (C6), 26.1 (C7), 25.5 (C11), 21.1 (C15), 13.5 (C18).

Data were in accordance with the literature.[S6]

Sodium-3-β-estrone sulfate or Sodium (8*R*,9*S*,13*S*,14*S*)-13-methyl-17-oxo-7,8,9,11,12,13,14,15,16,17-decahydro-6*H*-cyclopenta[*a*]phenanthren-3-yl sulfate (**17**)[S7]

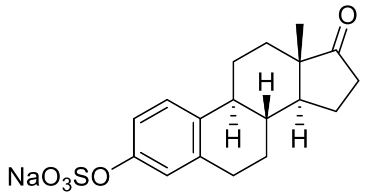

Following **general procedure 1**: β-Estrone (**15**) (270.4 mg, 1.0 mmol) and TBSAB (530.8 mg, 2.0 mmol) were dissolved in anhydrous MeCN (4.0 mL) and heated under reflux for 5h. After the completion of reaction, the flask was cooled to room temperature and the solvent removed under reduced pressure. The crude product was purified by **work up procedure B** to afford the title compound as white solid (269 mg, 72%).

M.P. 188-190 °C (dec.) (Lit. 226-228 °C)[S7]

^{1}H NMR (300 MHz, $CDCl_3$) δ 7.15 (dd, *J* = 8.4, 1.1 Hz, 1H), 6.64 (dd, *J* = 8.4, 2.8 Hz, 1H), 6.59 (d, *J* = 2.6 Hz, 1H), 2.90-2.84 (m, 2H), 2.51 (dd, *J* = 18.4, 8.4 Hz, 1H), 2.44–2.33 (m, 1H), 2.31–1.85 (m, 5H), 1.78–1.30 (m, 6H), 0.91 (s, 3H).

^{13}C NMR (101 MHz, $CDCl_3$) δ 221.3, 153.6, 138.2, 132.2, 126.6, 115.4, 112.9, 50.5, 48.1, 44.0, 38.4, 36.0, 31.7, 29.6, 26.6, 26.0, 21.7, 14.0.

LRMS. *m/z* (ES^-) 349.11 ($[M-Na]^-$, 100%), 350.11 ($[M^{13}C-Na]^-$, 10%).

HRMS. *m/z* (ES^-) $C_{18}H_{21}O_5S$ requires 349.1110, found 349.1120 $[M-Na]^-$.

Data were in accordance with the literature.[S7]

16-d_2-β-estrone or (8*R*,9*S*,13*S*,14*S*)-3-hydroxy-13-methyl-6,7,8,9,11,12,13,14,15,16-decahydro-17*H*-cyclopenta[*a*]henanthrene-17-one-16,16-D_2 (**18**)[S8]

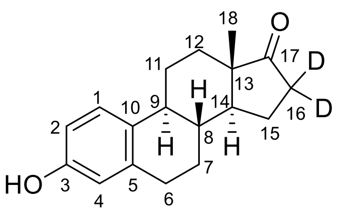

A round bottom flask was charged with estrone (**15**) (270.4 mg, 1.0 mmol), Sodium deuteroxide (0.2 mL) were dissolved in MeOD (8 mL) under an inert environment. The reaction mixture was heated under reflux for 24 h. After the completion of reaction, the flask was cooled to room temperature and 10% sulfuric acid was added dropwise to achieve pH 7. The white precipitate was collected by filtration, washed with distilled water (10 mL), and crystallized in ethanol (15 mL). The solvent was removed under reduced pressure to afford the desired deuterium-labelled of estrone as white solid (240 mg, 88%).

M.P. 201-203 °C (Lit. not stated)

^{1}H NMR (400 MHz, DMSO-d$_6$) δ 9.01 (s, 1H, C3-OH), 7.08–7.01 (m, 1H, C1-H), 6.51 (dd, *J* = 8.4, 2.7 Hz, 1H, C2-H), 6.45 (d, *J* = 2.6 Hz, 1H, C4-H), 2.84–2.67 (m, 2H), 2.34–2.26 (m, 1H, C11-H), 2.13 (s, 1H, C9-H), 1.94 (t, *J* = 4.5 Hz, 1H, C15-H), 1.89 (dd, *J* = 7.3, 4.5 Hz, 1H, C7-H), 1.74 (dd, *J* = 8.9, 2.6 Hz, 1H, C12-H), 1.58–1.43 (m, 3H), 1.39–1.27 (m, 3H), 0.82 (s, 3H, C18-CH$_3$).

^{13}C NMR (101 MHz, DMSO-d$_6$) δ 219.8 (C17), 155.0 (C3), 137.1(C5), 129.9 (C10), 126.0 (C1), 114.9 (C4), 112.8 (C2), 49.5 (C14), 47.3 (C13), 43.4 (C9), 37.9 (C8), 34.9 (C16), 31.3 (C12), 29.0 (C6), 26.1 (C7), 25.5 (C11), 20.9 (C15), 13.5 (C18).

LRMS. *m/z* (TOF MS APAP$^+$) 271.17 ([M-$^2H_2$+$^1H_3$]$^+$, 100%), 272.18 (M^{13}C-$^2H_2$+$^1H_3$]$^+$, 80%), 273.18 ([M-$^2H_1$+$^1H_2$]$^+$, 20%).

HRMS. *m/z* (TOF MS APAP$^+$) $C_{18}{}^1H_{21}{}^2H_2O_2$ requires 273.1824, found 273.1806 [M-$^2H_1$+$^1H_2$]$^+$,

It should be noted that a 72% level of $^2H_2$ deuterium incorporation was validated by ^{1}H NMR analysis due to the disappearance of C(16) signals indicating $^2H_2$ was present. Due to the exchangeability of the C(16)-$^2H_2$ protons with ^{1}H a mixture of un-deuterated and partially deuterated estrone was observed. Figure S1 shows the theoretical versus observed mass spectra for all possible deuteration scenarios.

16-d$_2$-Sodium-3-β-estrone sulfate or Sodium (8*R*,9*S*,13*S*,14*S*)-13-methyl-17-oxo-7,8,9,11,12,13,14,15,16,17-decahydro-6*H*-cyclopenta[*a*]phenanthren-3-yl-16,16-d2 sulfate (**20**)

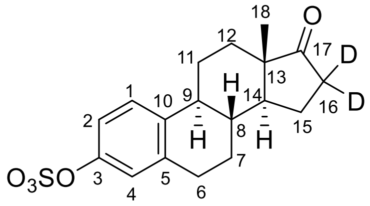

Following **general procedure 1**: Estrone-d$_2$ (**18**) (136 mg, 0.5 mmol) and TBSAB (265 mg, 1.0 mmol) were dissolved in anhydrous MeCN (2.0 mL) and heated under reflux for 7 h. After the completion of reaction, the flask was cooled to room temperature and the solvent removed under reduced pressure. The crude product was purified by **work up procedure B** and washed with 10 mL of MeCN to afford the title compound as yellow solid (149 mg, 78%).

M.P. 210-212 °C

^{1}H NMR (400 MHz, DMSO-d$_6$) δ 7.16 (d, *J* = 8.3 Hz, 1H, C1-H), 6.91 – 6.86 (m, 2H, C2-H, C4-H), 2.85–2.76 (m, 2H), 2.39–2.32 (m, 1H, C11-H), 2.24-2.16 (m, 1H, C9-H), 1.99–1.89 (m, 2H, C15-H), 1.76 (dd, *J* = 8.7, 2.6 Hz, 1H, C12-H), 1.60–1.46 (m, 3H), 1.43–1.32 (m, 3H), 0.83 (s, 3H).

^{13}**C NMR** (101 MHz, DMSO-d_6) δ 219.8 (C17), 151.29 (C3), 136.60 (C5), 134.31 (C10), 125.5 (C1), 120.5 (C4), 118.1 (C2), 49.5 (C14), 47.3 (C13), 43.6 (C9), 37.7 (C8), 35.4 (C16), 31.3 (C12), 29.0 (C6), 26.0 (C7), 25.5 (C11), 20.9 (C15), 13.4 (C18).

LRMS. *m/z* (ESI^-) 351.12 ($[M\text{-}Na]^-$, 100%), 350.1176 ($[M\text{-}^2H_1\text{+}^1H_1\text{-}Na]^-$, 50%), 352.12 ($[M^{13}C\ \text{-}Na]^-$, 20%), 353.12 (10%), 349.11 (10%).

HRMS. *m/z* (ES^-) $C_{18}{}^1H_{19}{}^2H_2O_5S$ requires 351.1235, found 351.1243 $[M\text{+}^2H_2]^-$.

^{1}H NMR analysis confirmed a 67% level of $^2H_2$ incorporation. Mass spectrometric comparison of theoretical deuteration levels versus the observed is shown in Figure S2.

Copies of ^{1}H and ^{13}C NMR spectra

^{1}H NMR spectrum of **3** (400 MHz, DMSO-d_6)

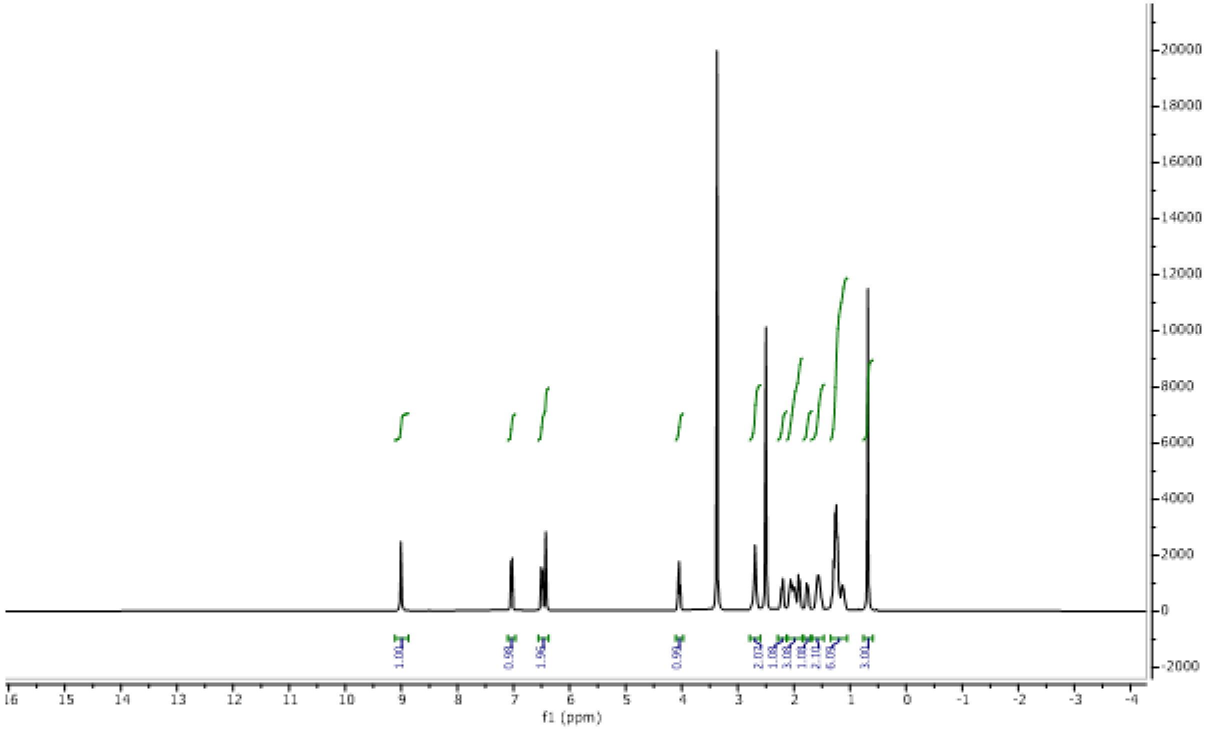

^{13}C NMR spectrum of **3** (101 MHz, DMSO-d_6)

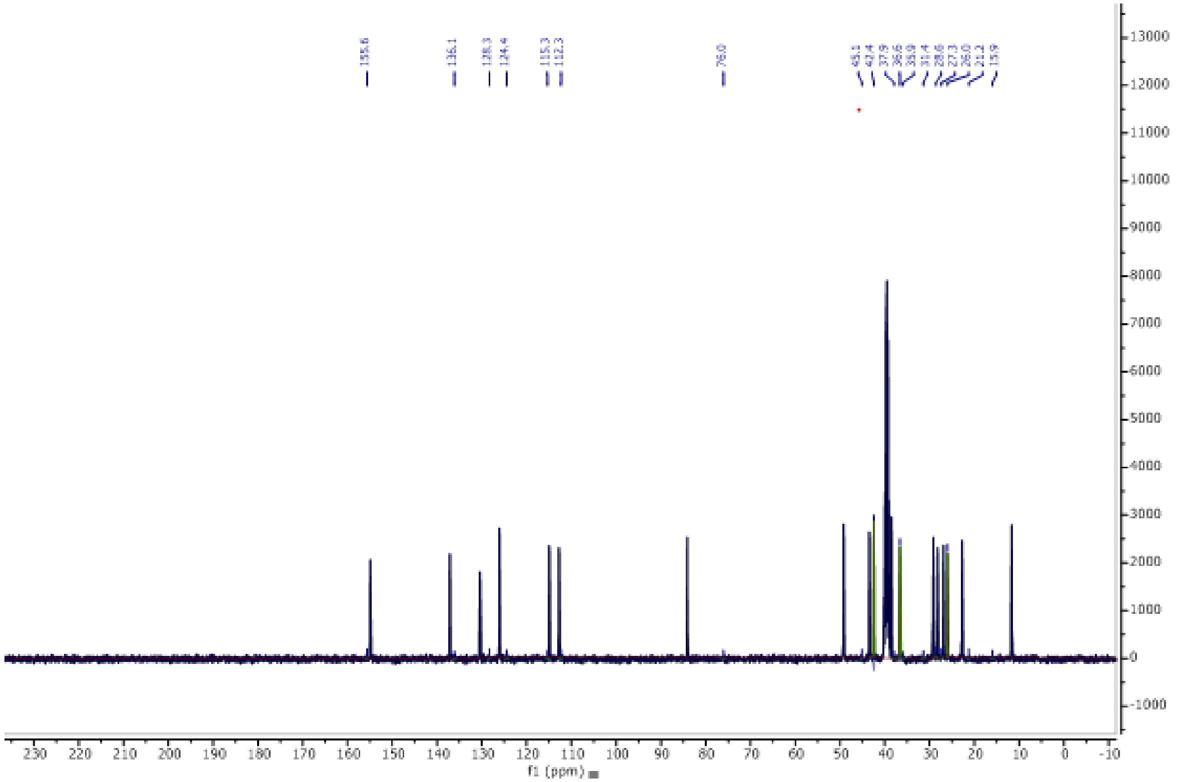

^{1}H NMR spectrum of **5** (400 MHz, DMSO-d$_6$)

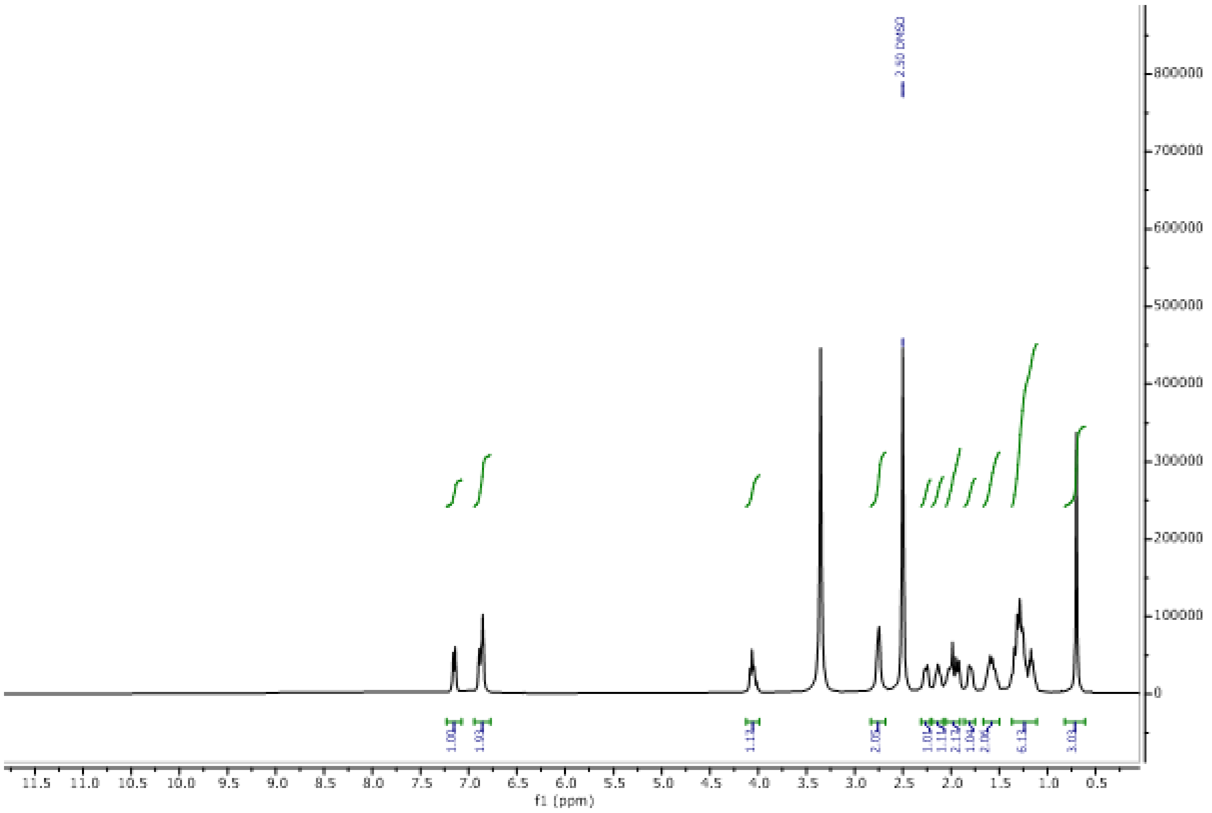

^{13}C NMR spectrum of **5** (101 MHz, DMSO-d_6)

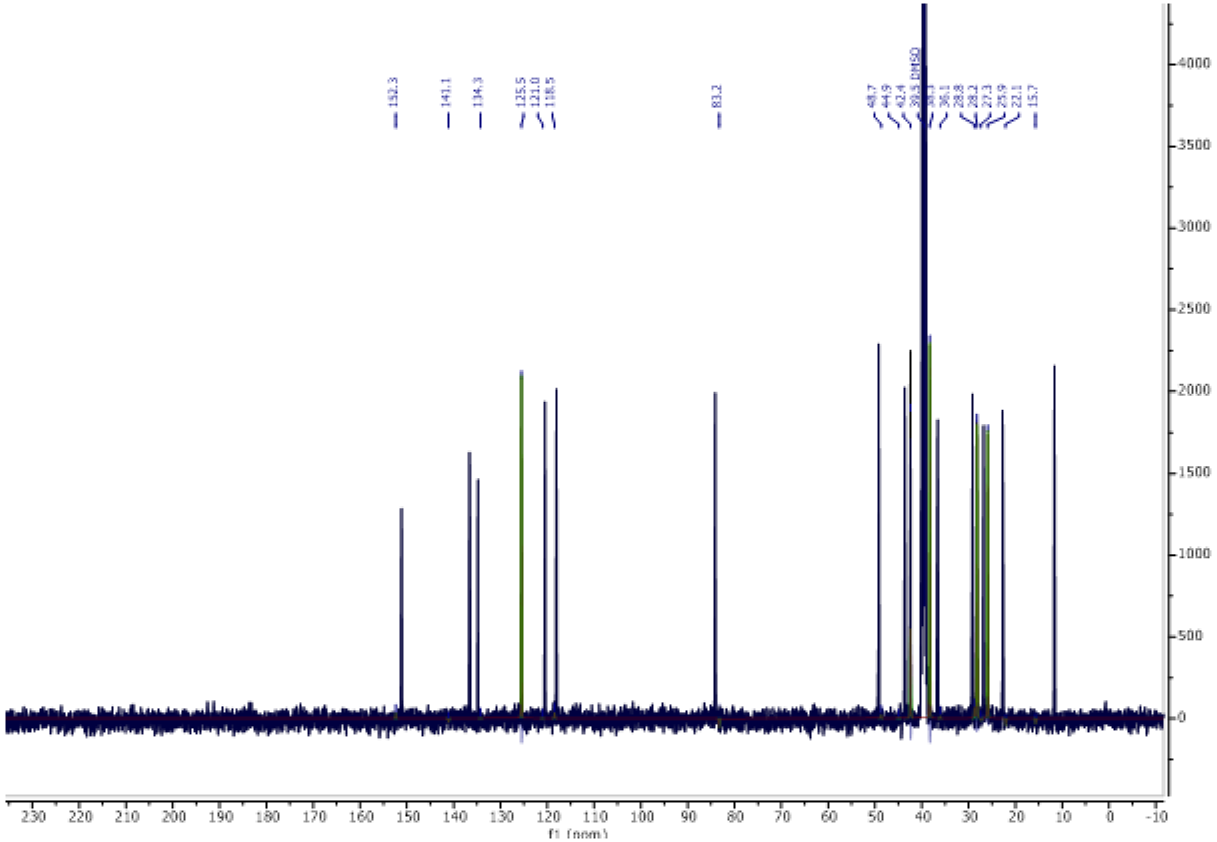

^{1}H NMR spectrum of **8** (400 MHz, DMSO-d_6)

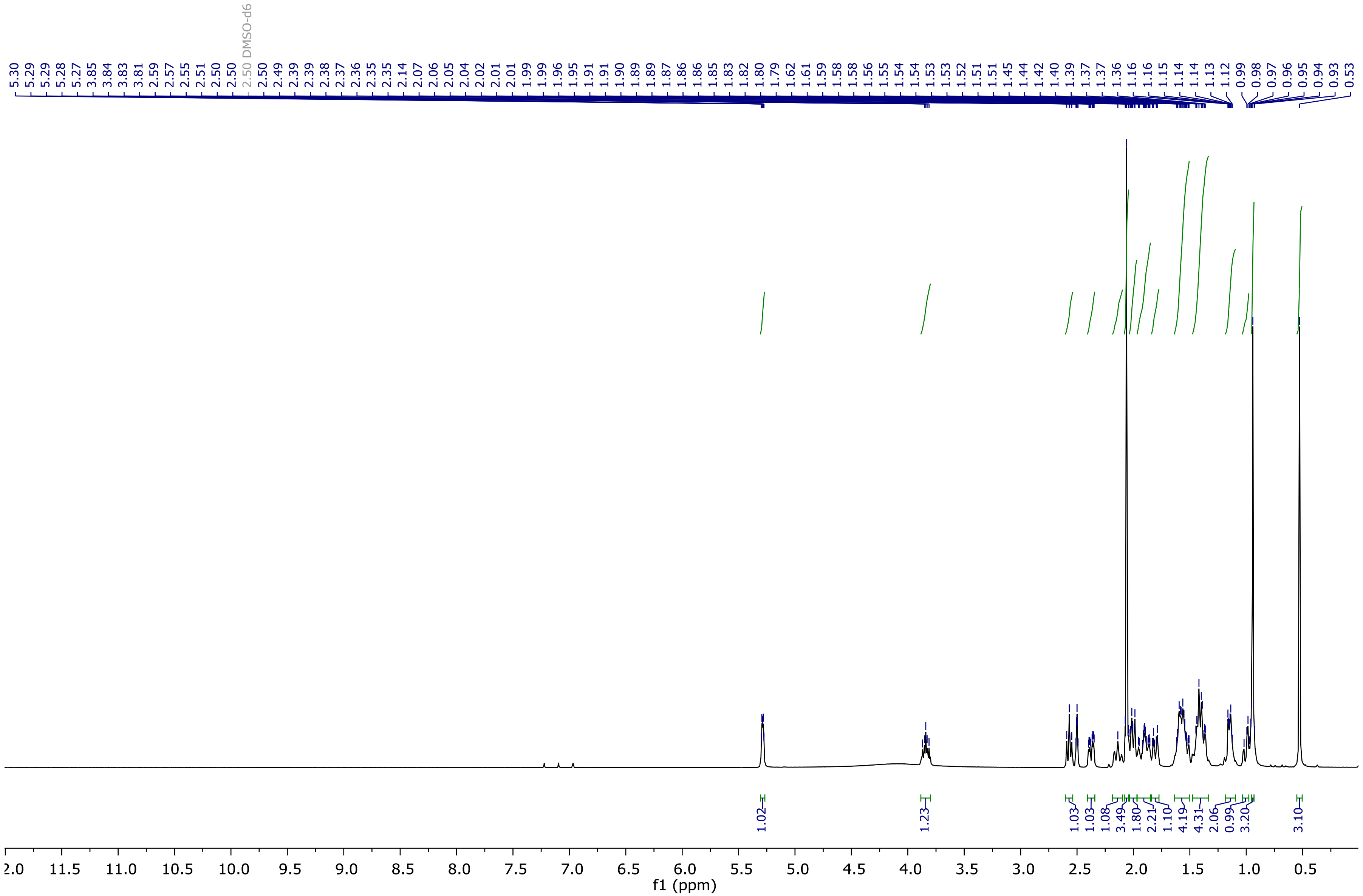

^{13}C NMR spectrum of **8** (101 MHz, DMSO-d_6)

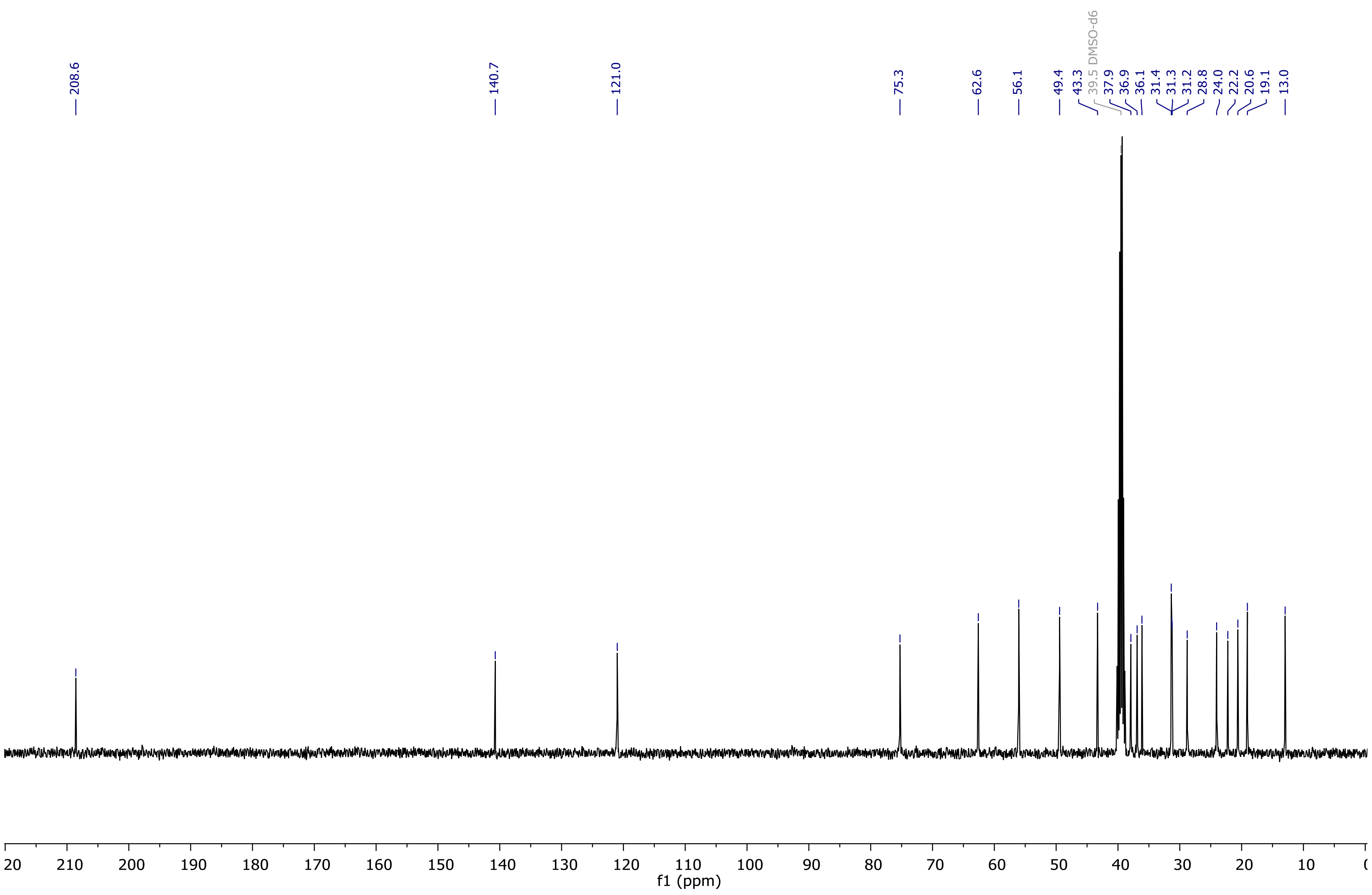

^{1}H NMR spectrum of **9** (400 MHz, DMSO-d_6)

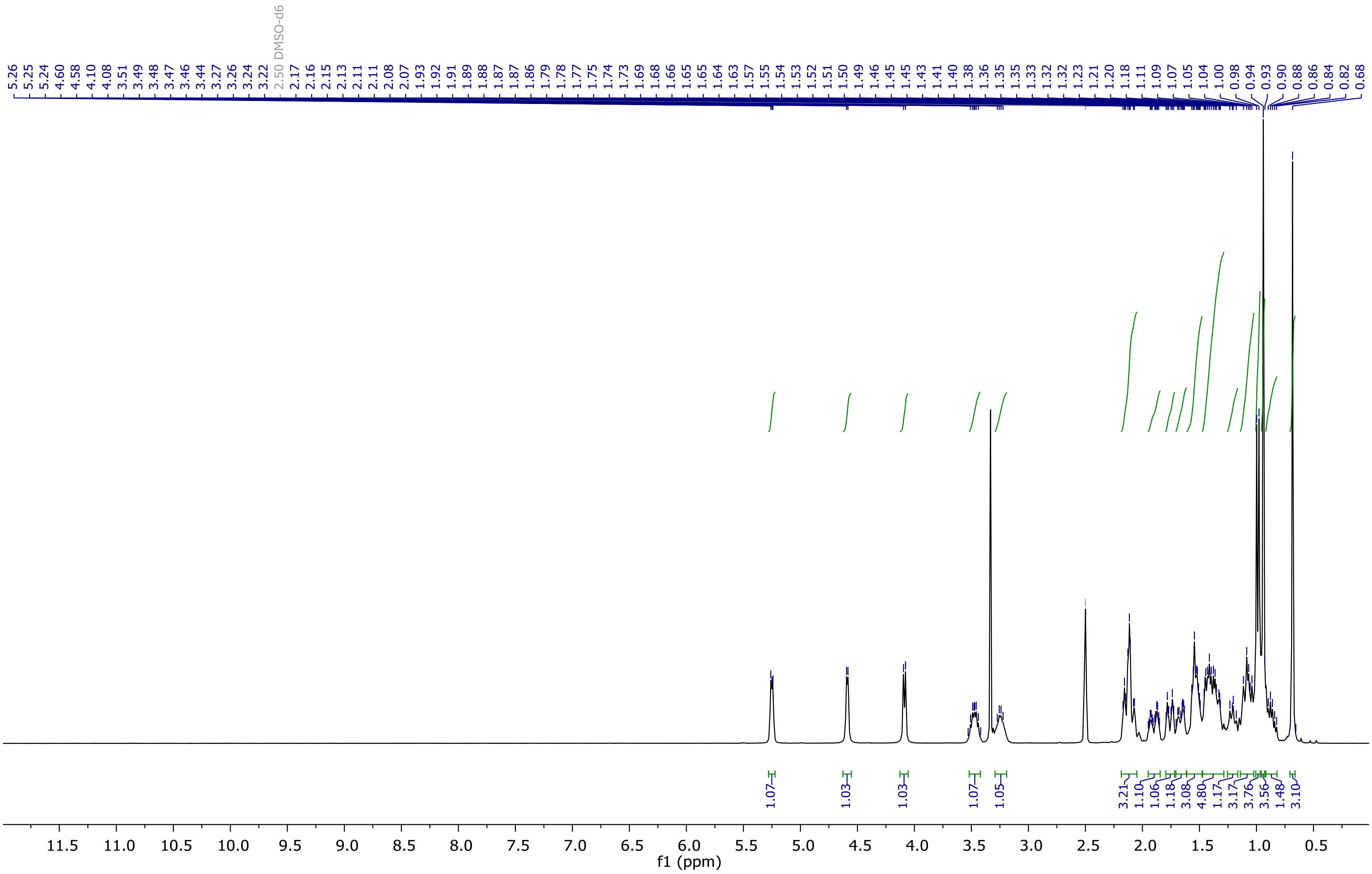

^{13}C NMR spectrum of **9** (101 MHz, DMSO-d$_6$)

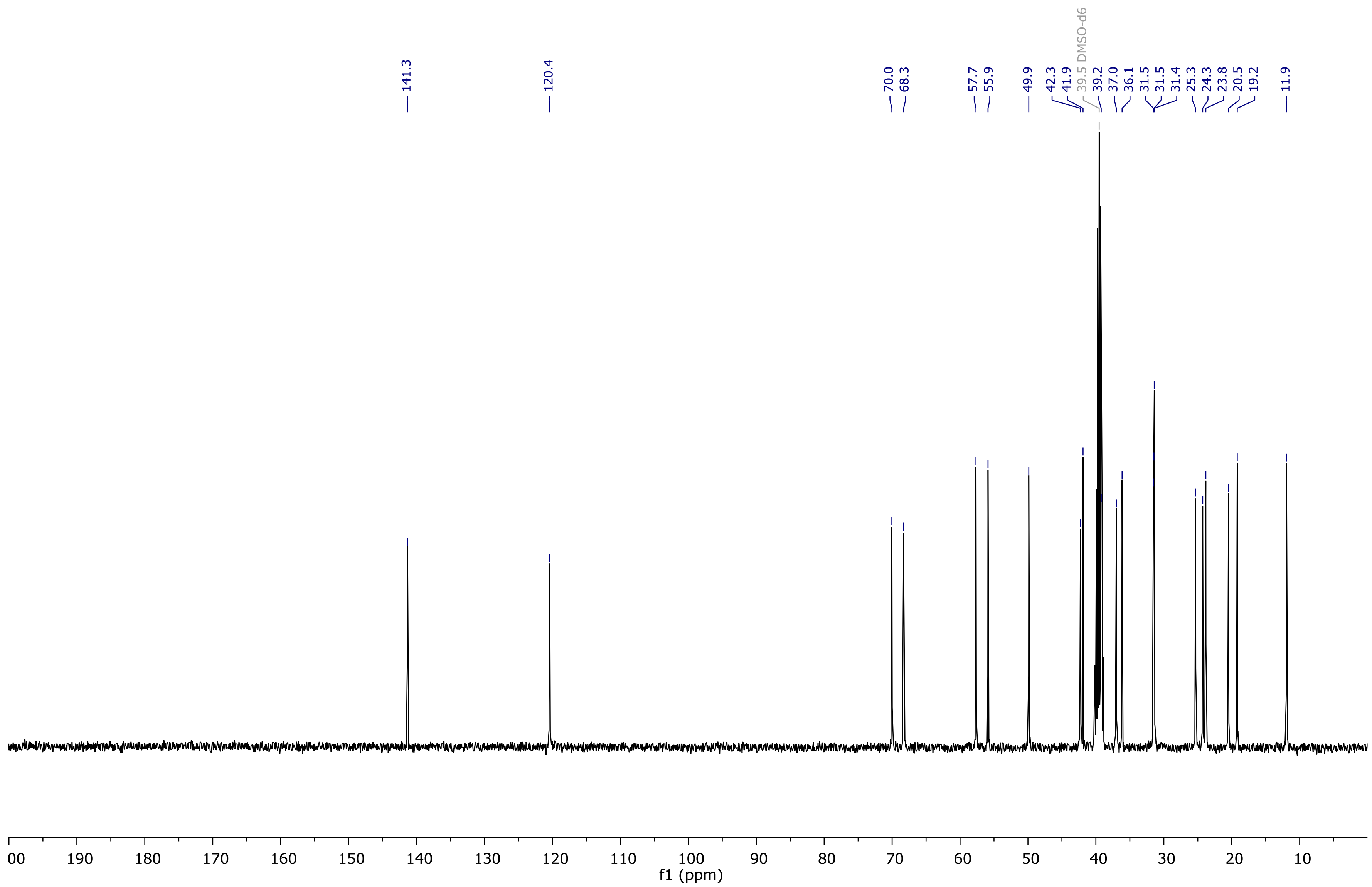

^{1}H NMR spectrum of **11** (400 MHz, DMSO-d_6)

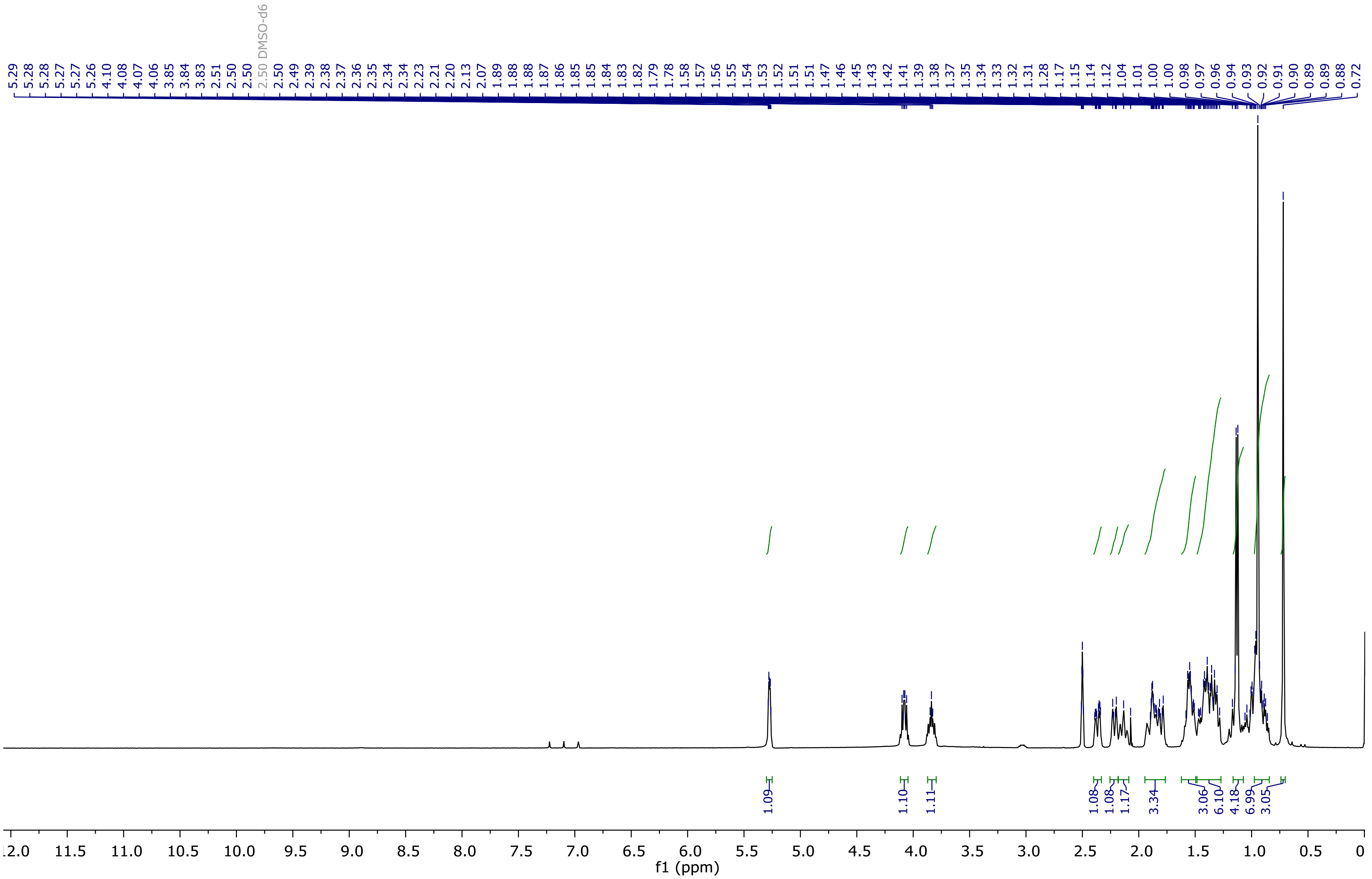

^{13}C NMR spectrum of **11** (101 MHz, DMSO-d_6)

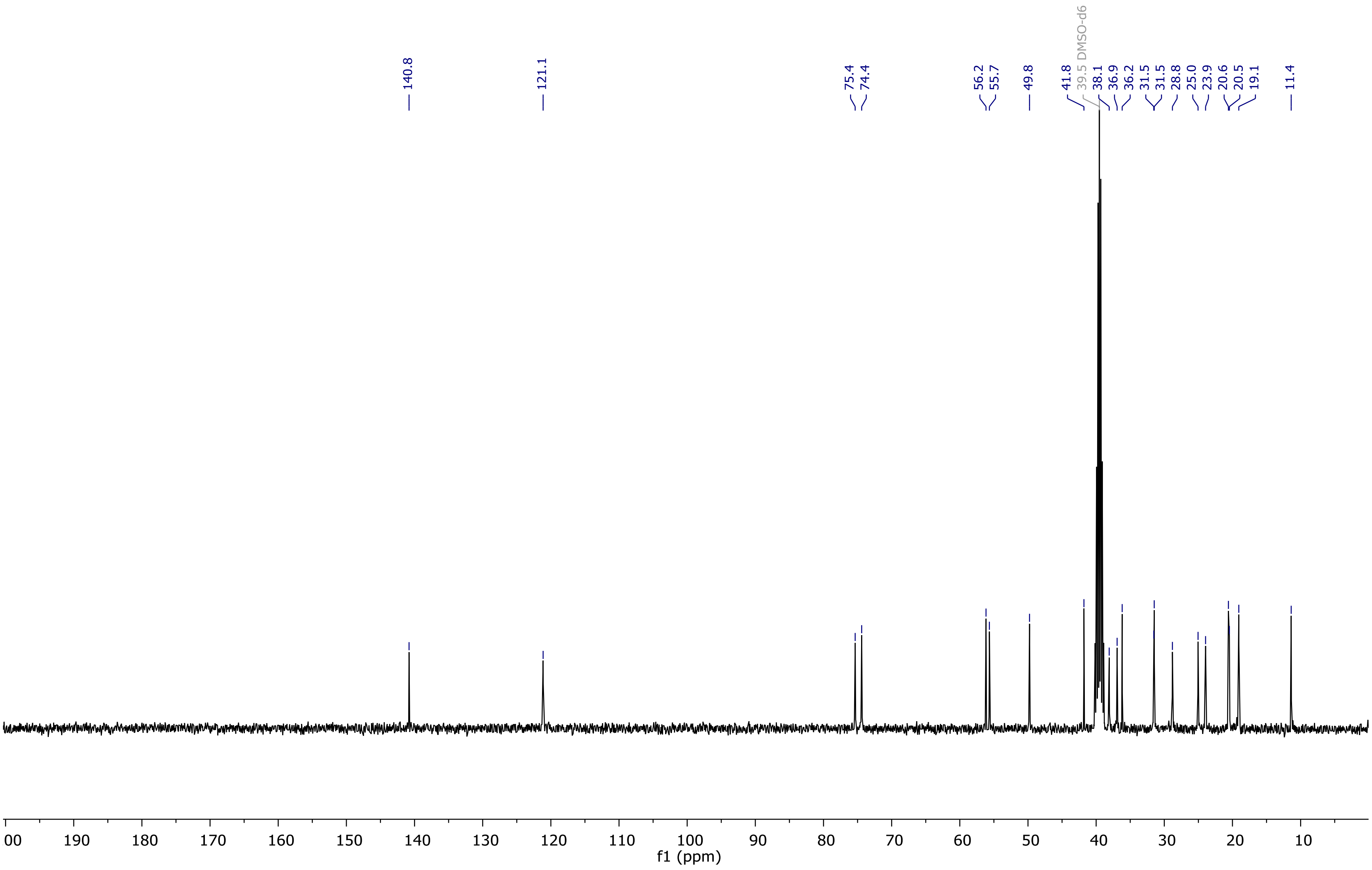

^{1}H-NMR spectrum of **14** (400 MHz, DMSO-d_6)

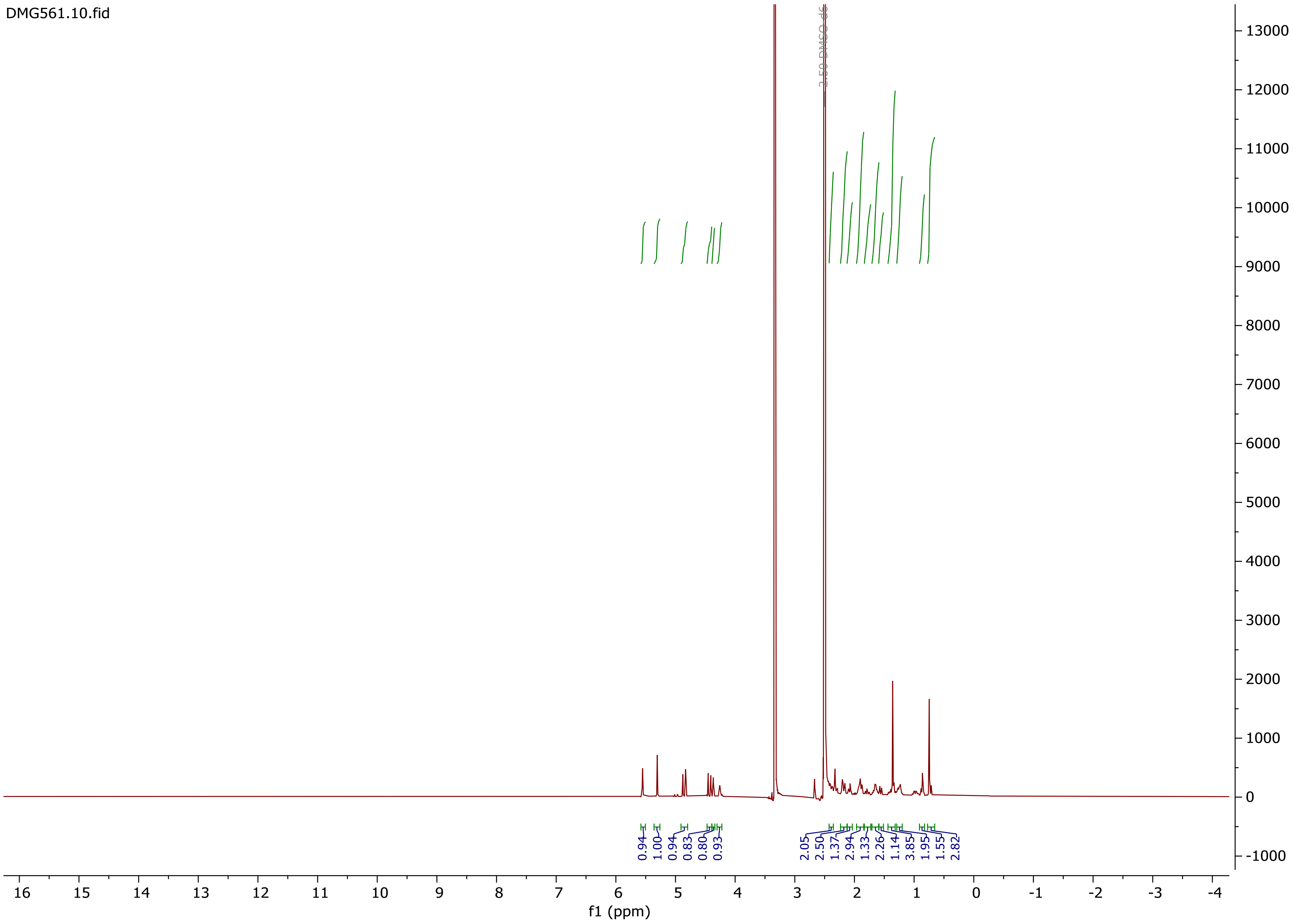

^{13}C NMR spectrum of **14** (101 MHz, DMSO-d_{6f})

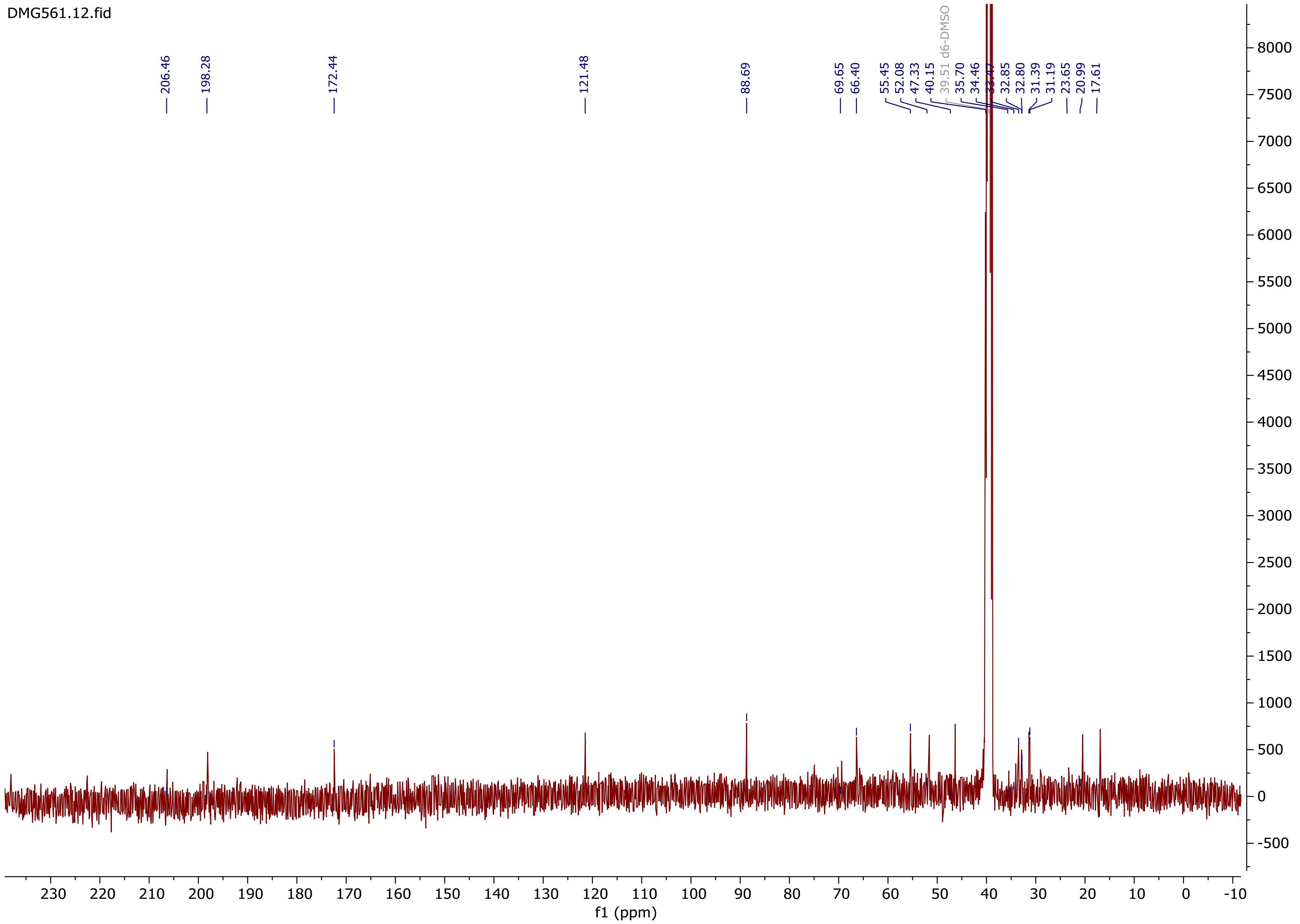

^{1}H NMR spectrum of **15** (400 MHz, DMSO-d_6)

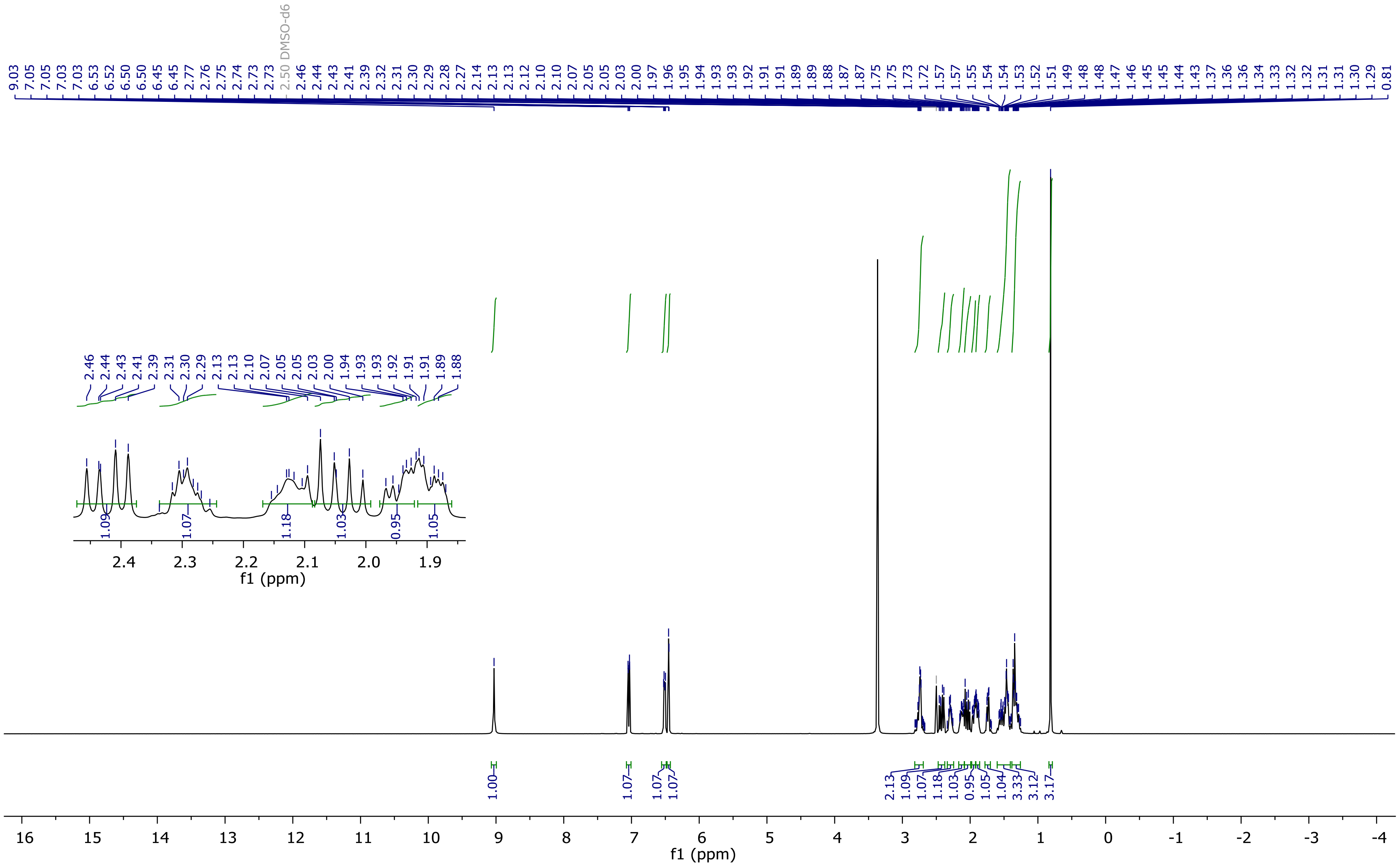

^{13}C NMR spectrum of **15** (101 MHz, DMSO-d$_6$)

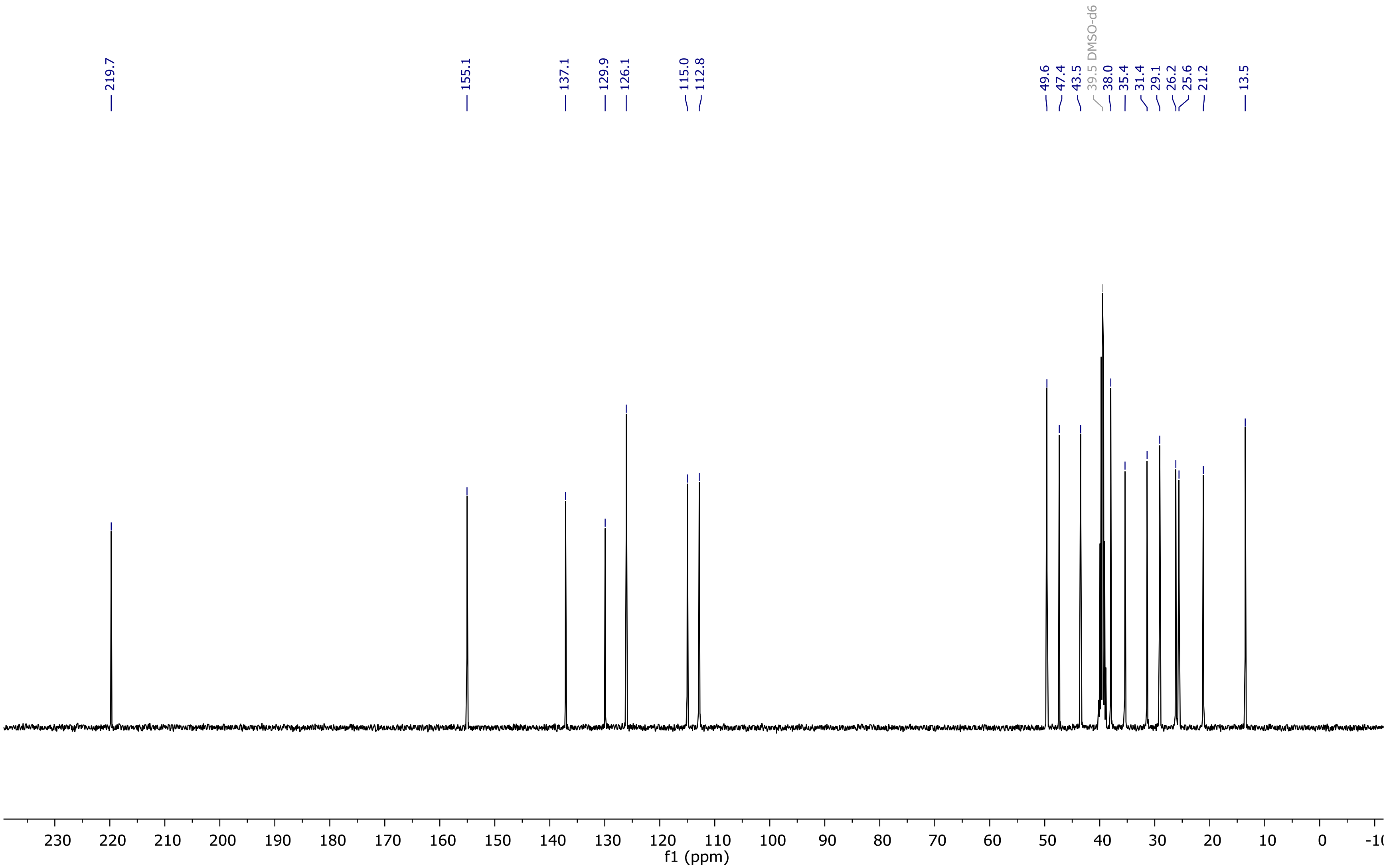

DEPT-45 spectrum of **15** (101 MHz, DMSO-d_6)

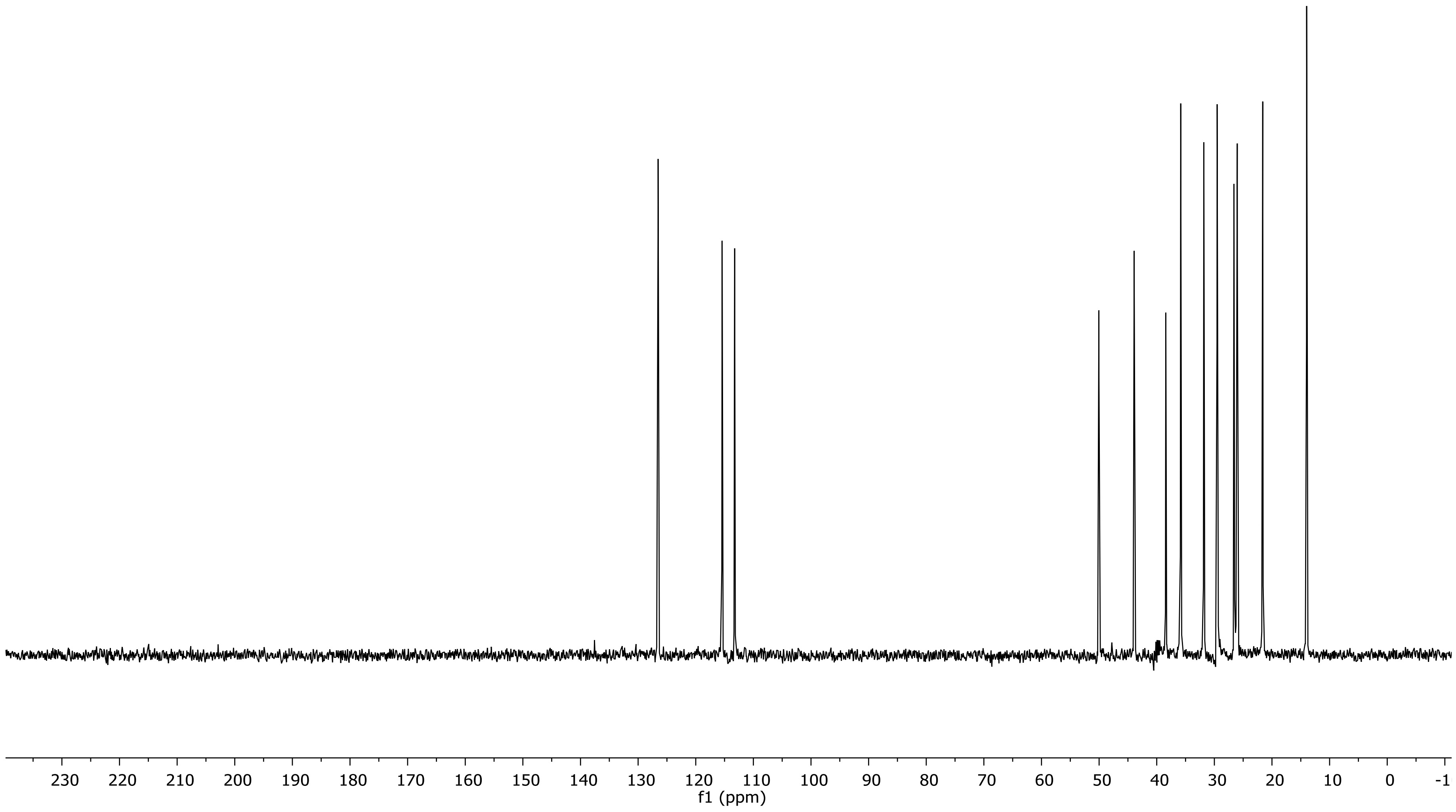

^{1}H-^{1}H COSY spectrum of **15** (101 MHz, DMSO-d_6)

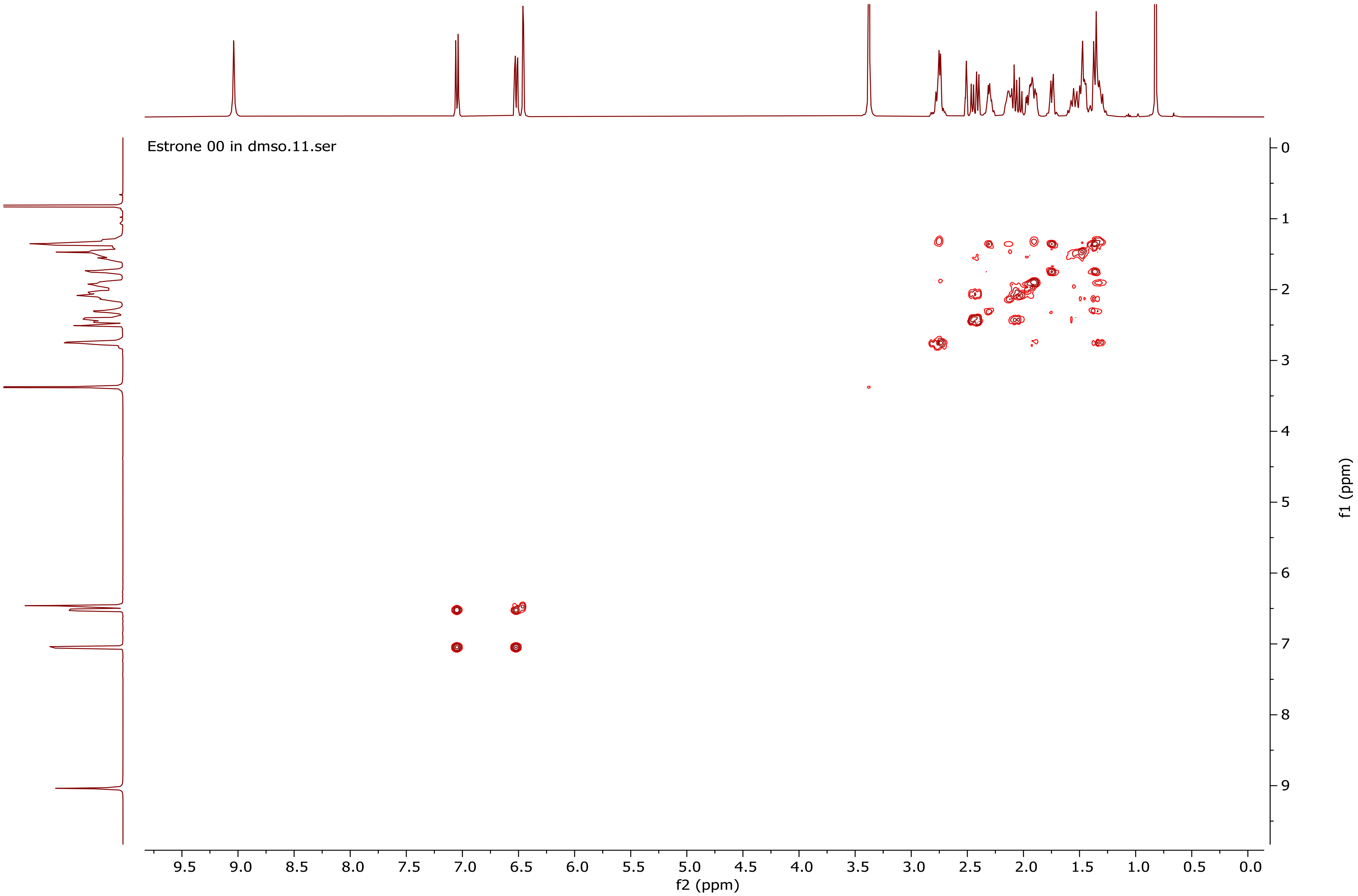

^{1}H-^{13}C HSQC spectrum of **15** (101 MHz, DMSO-d_6)

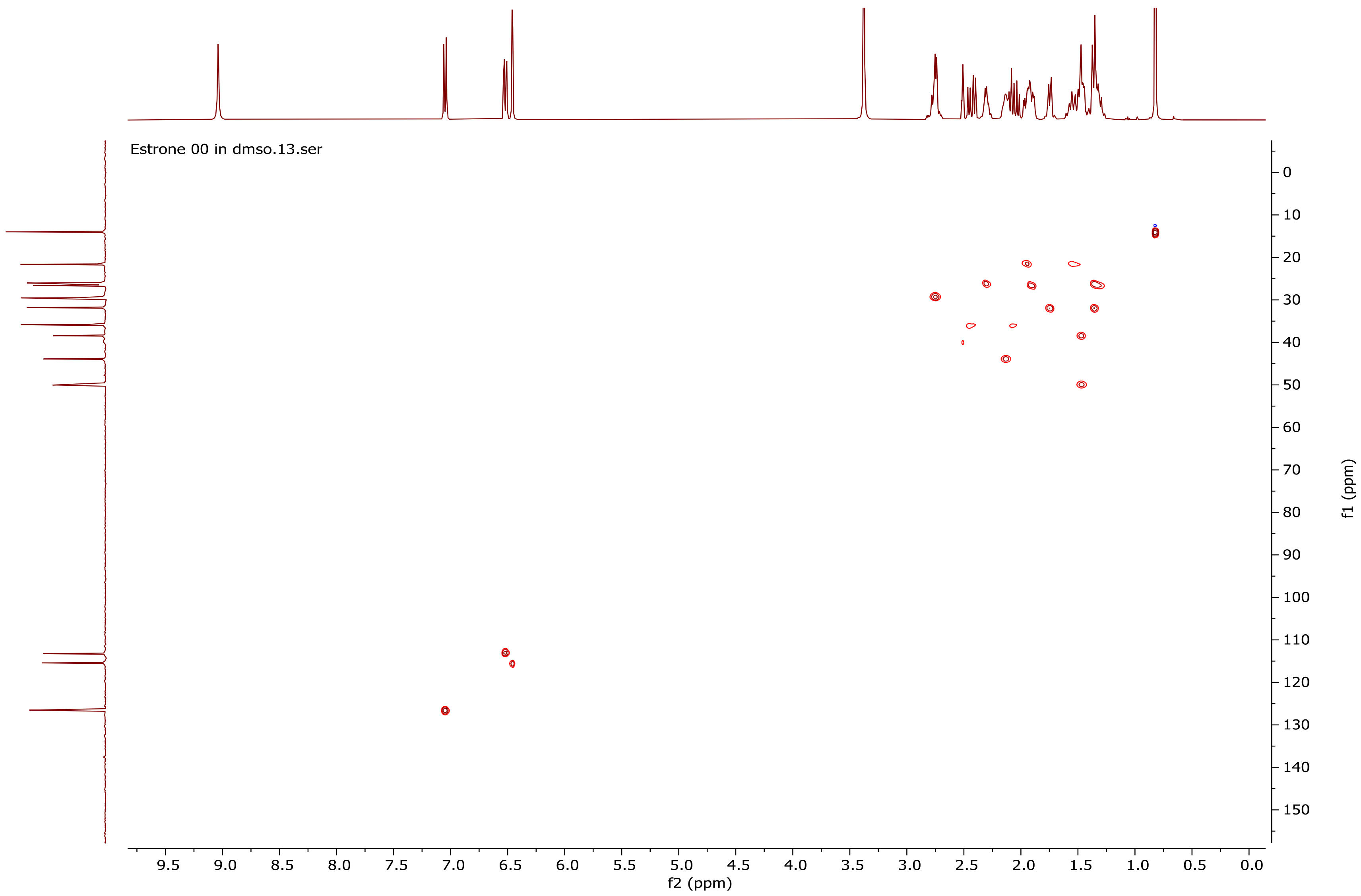

^{1}H-^{13}C HMBC spectrum of **15** (101 MHz, DMSO-d$_6$)

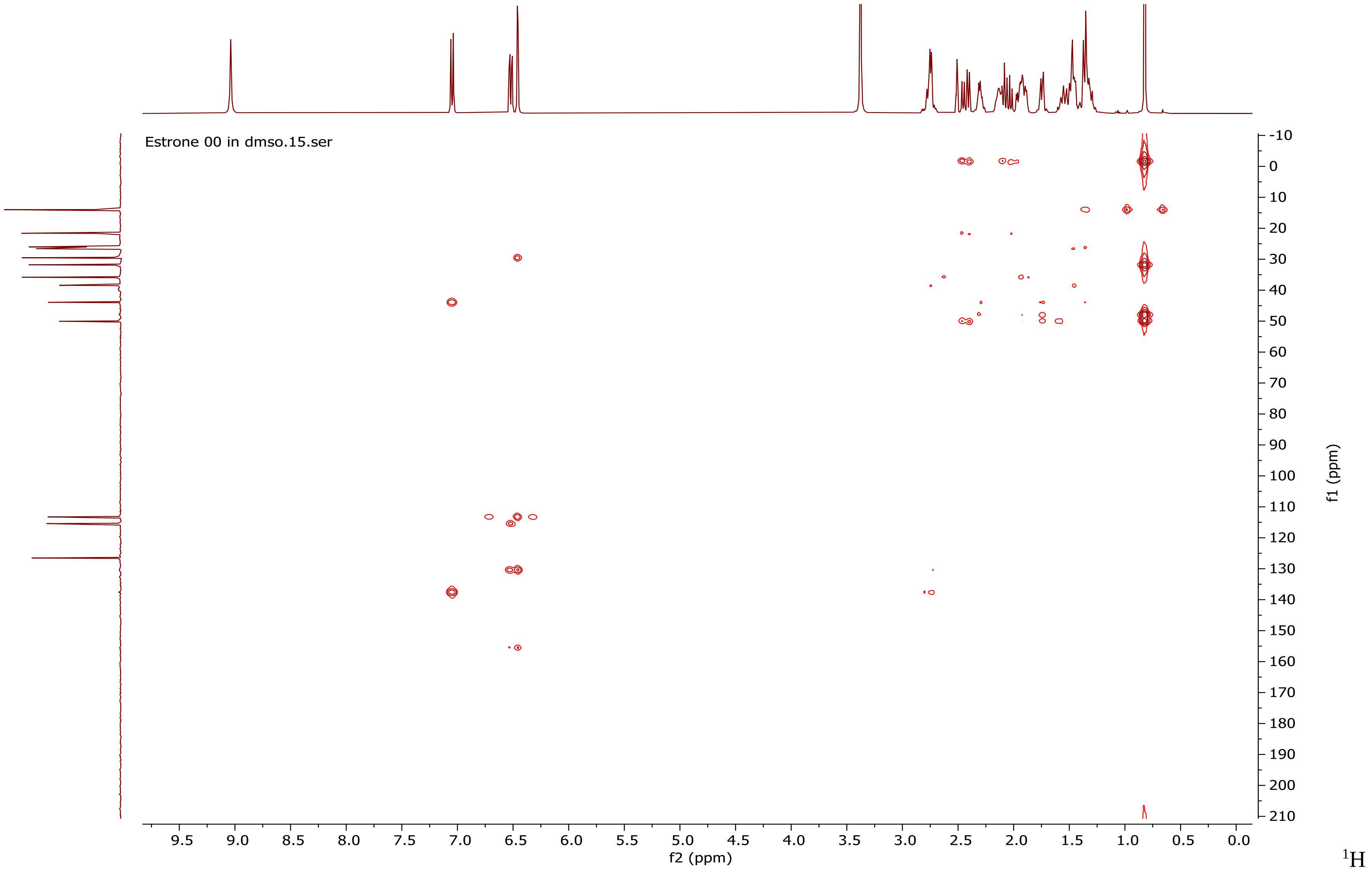

NMR spectrum of **17** (300 MHz, $CDCl_3$)

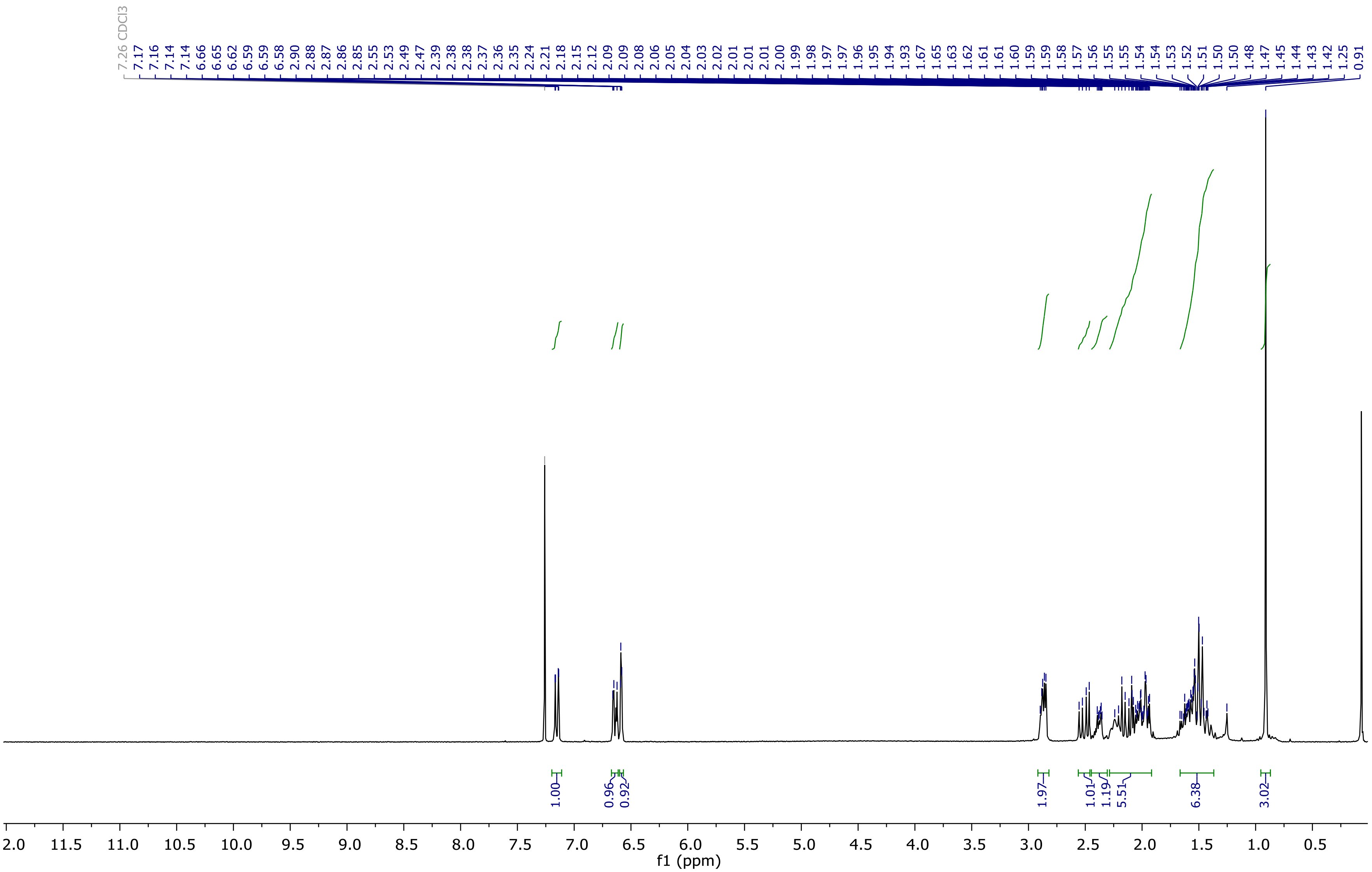

^{13}C NMR spectrum of **17** (300 MHz, $CDCl_3$)

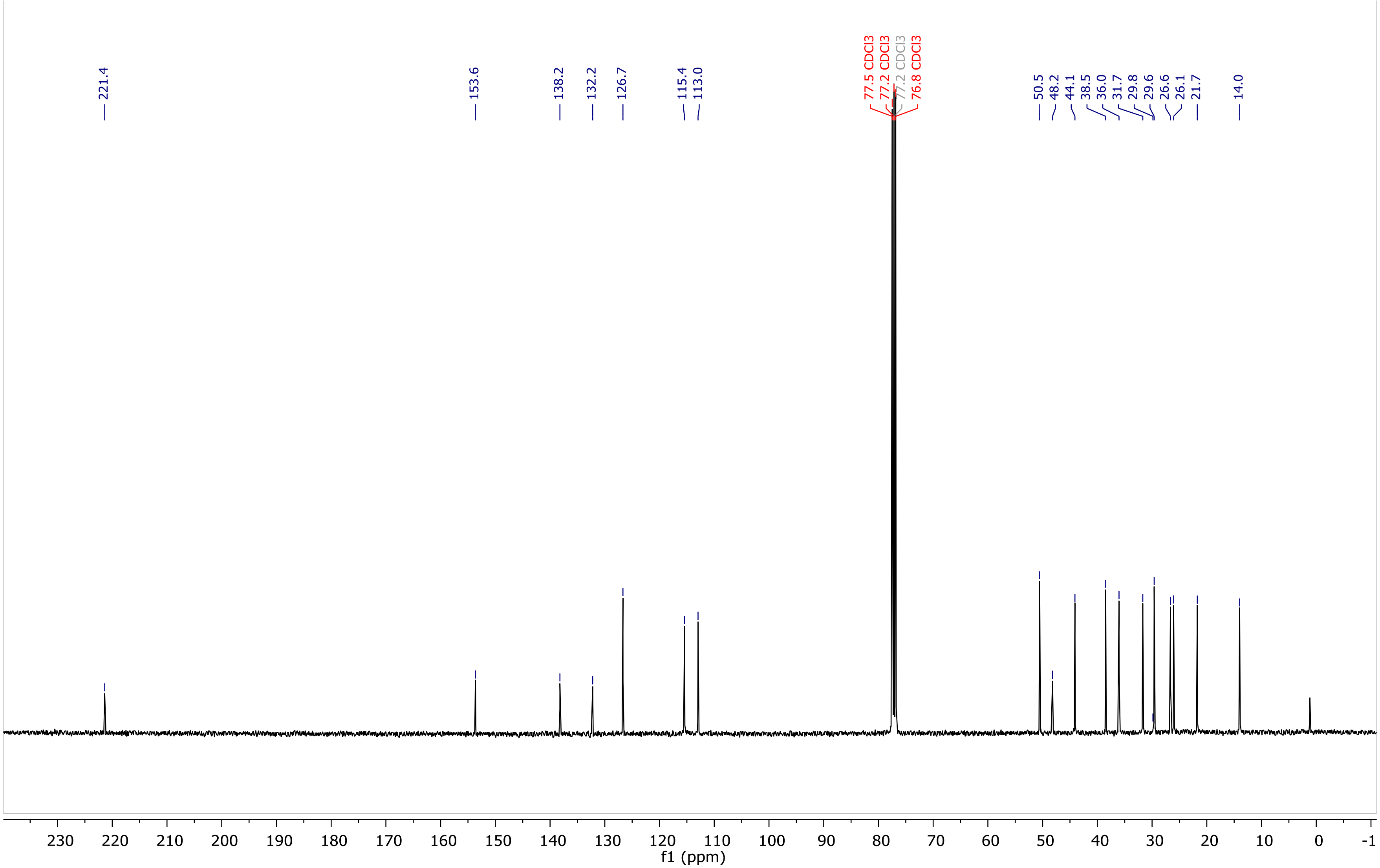

^{1}H NMR spectrum of **18** (400 MHz, DMSO-d_6)

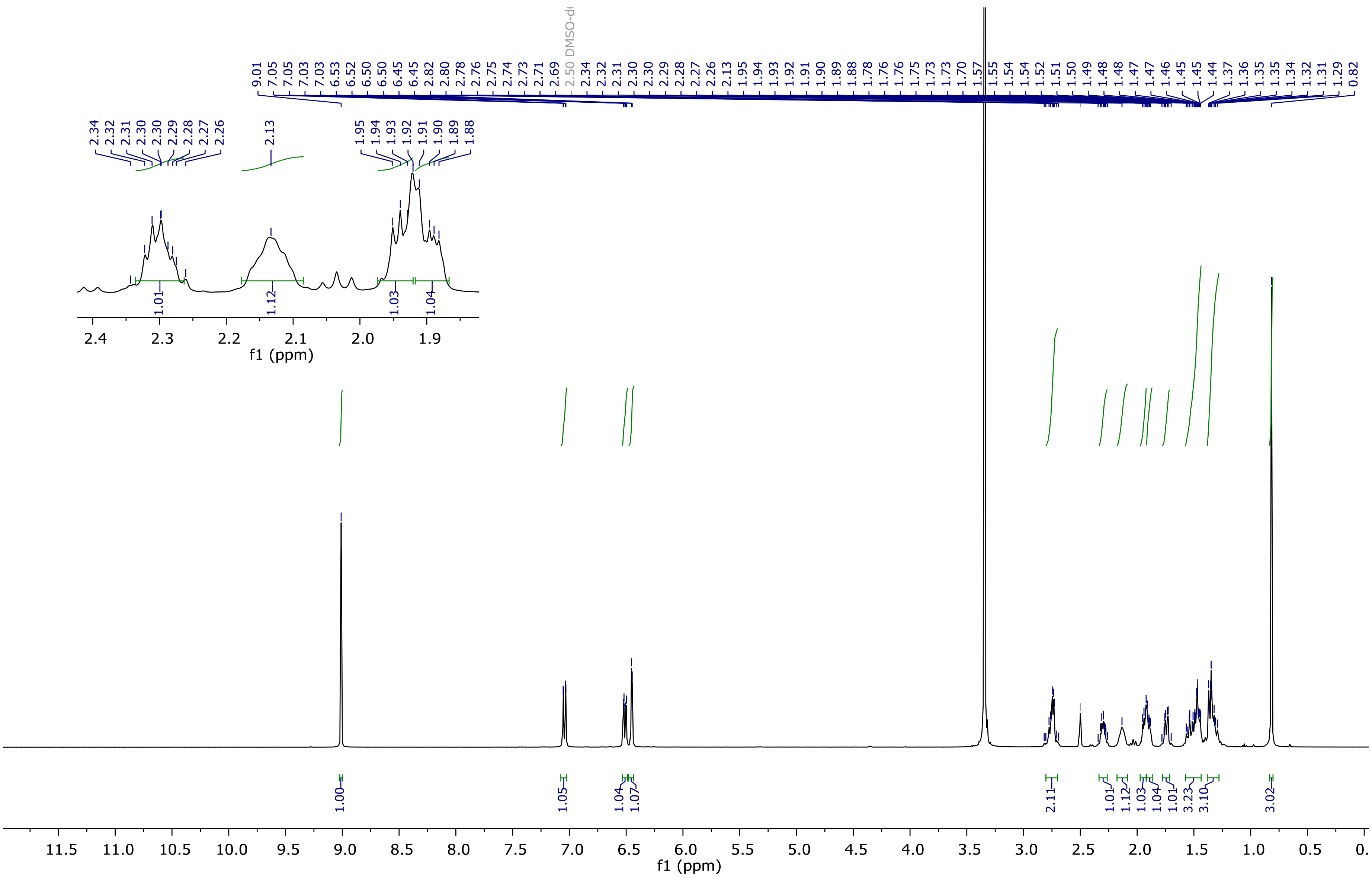

^{13}C NMR spectrum of **18** (101 MHz, DMSO-d_6)

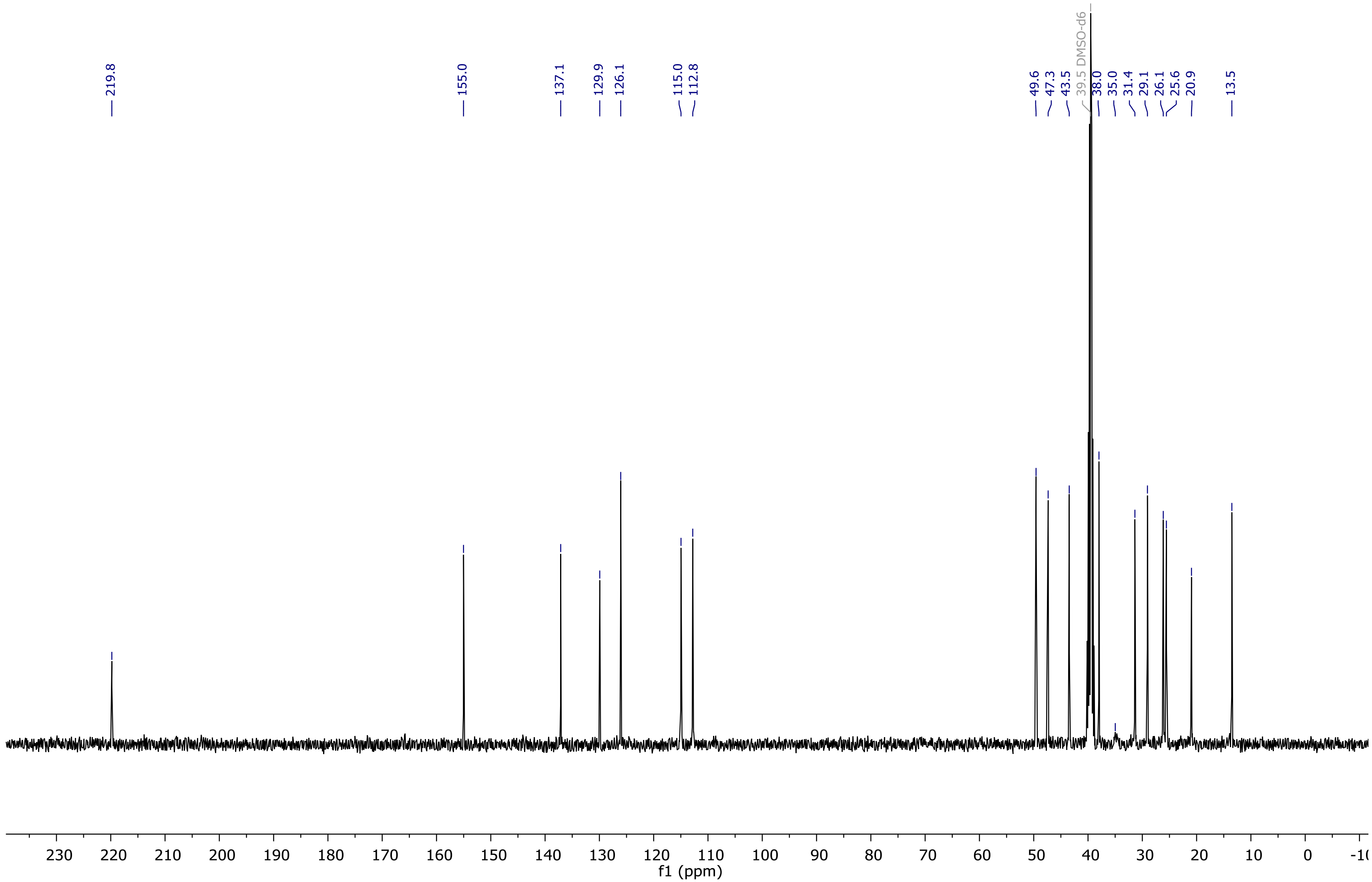

DEPT-45 spectrum of **18** (101 MHz, DMSO-d_6)

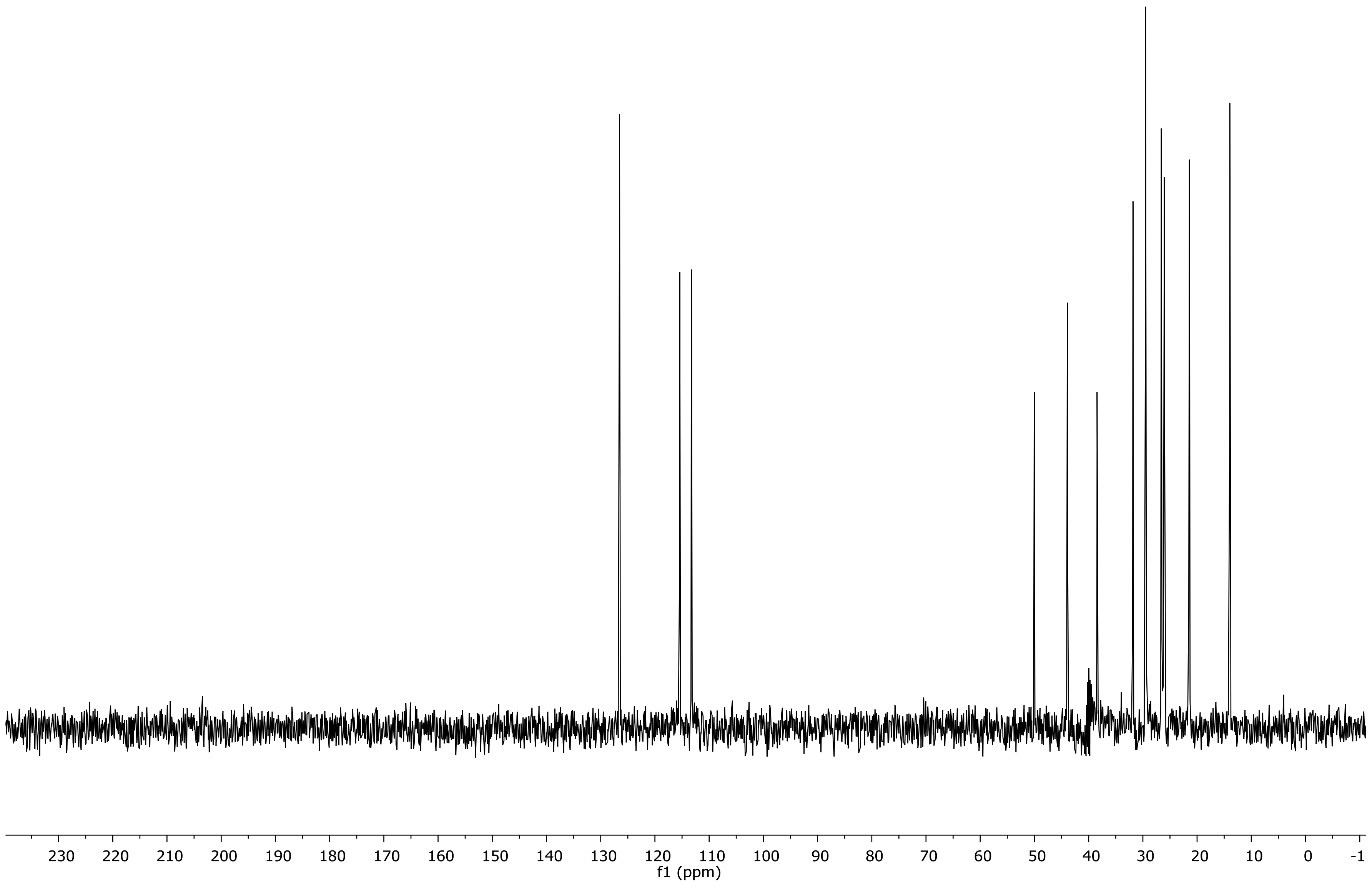

^{1}H-^{1}H COSY spectrum of **18** (101 MHz, DMSO-d$_6$)

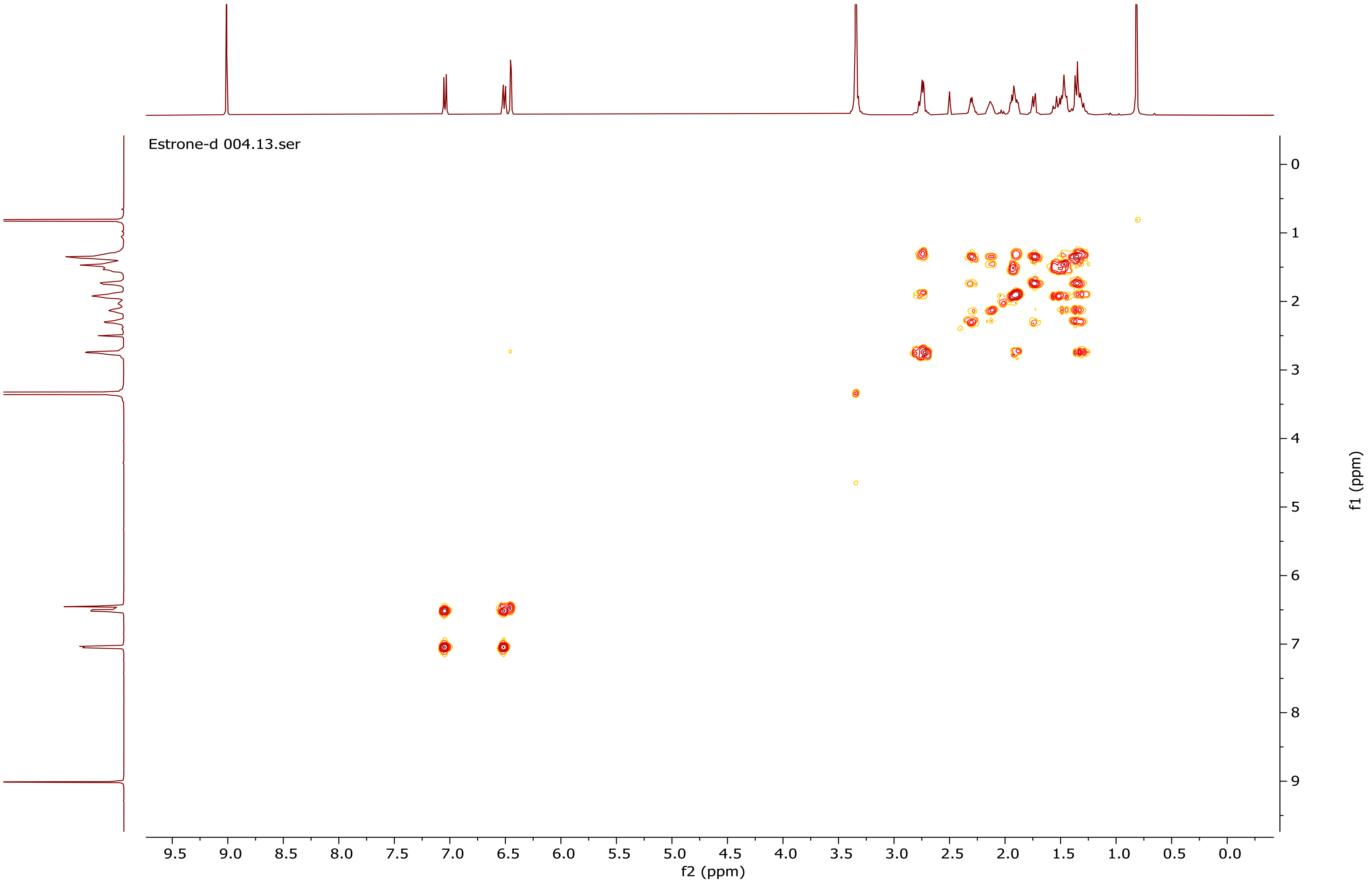

^{1}H-^{13}C HSQC spectrum of **18** (101 MHz, DMSO-d_6)

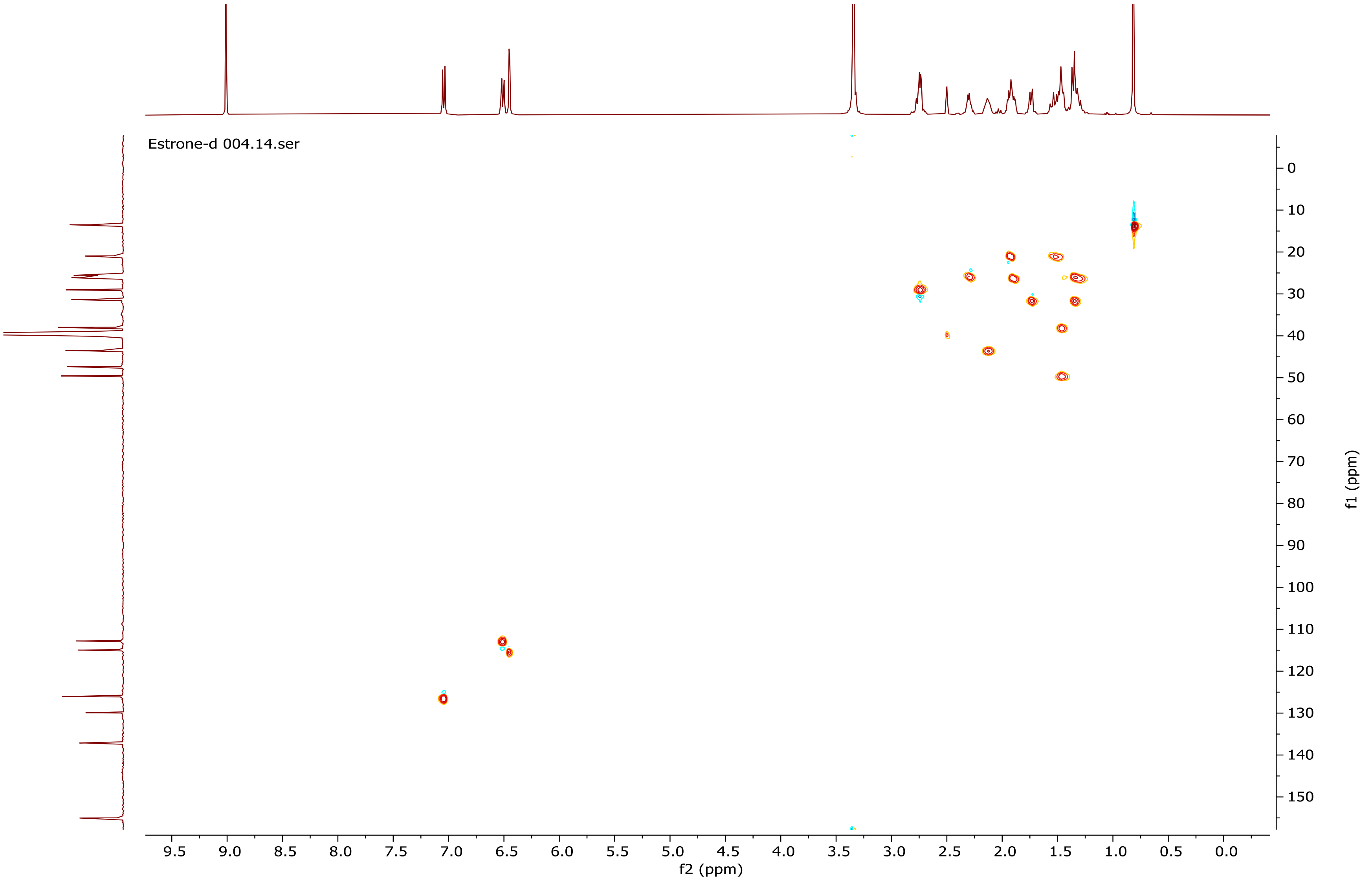

^{1}H-^{13}C HMBC spectrum of **18** (101 MHz, DMSO-d_6)

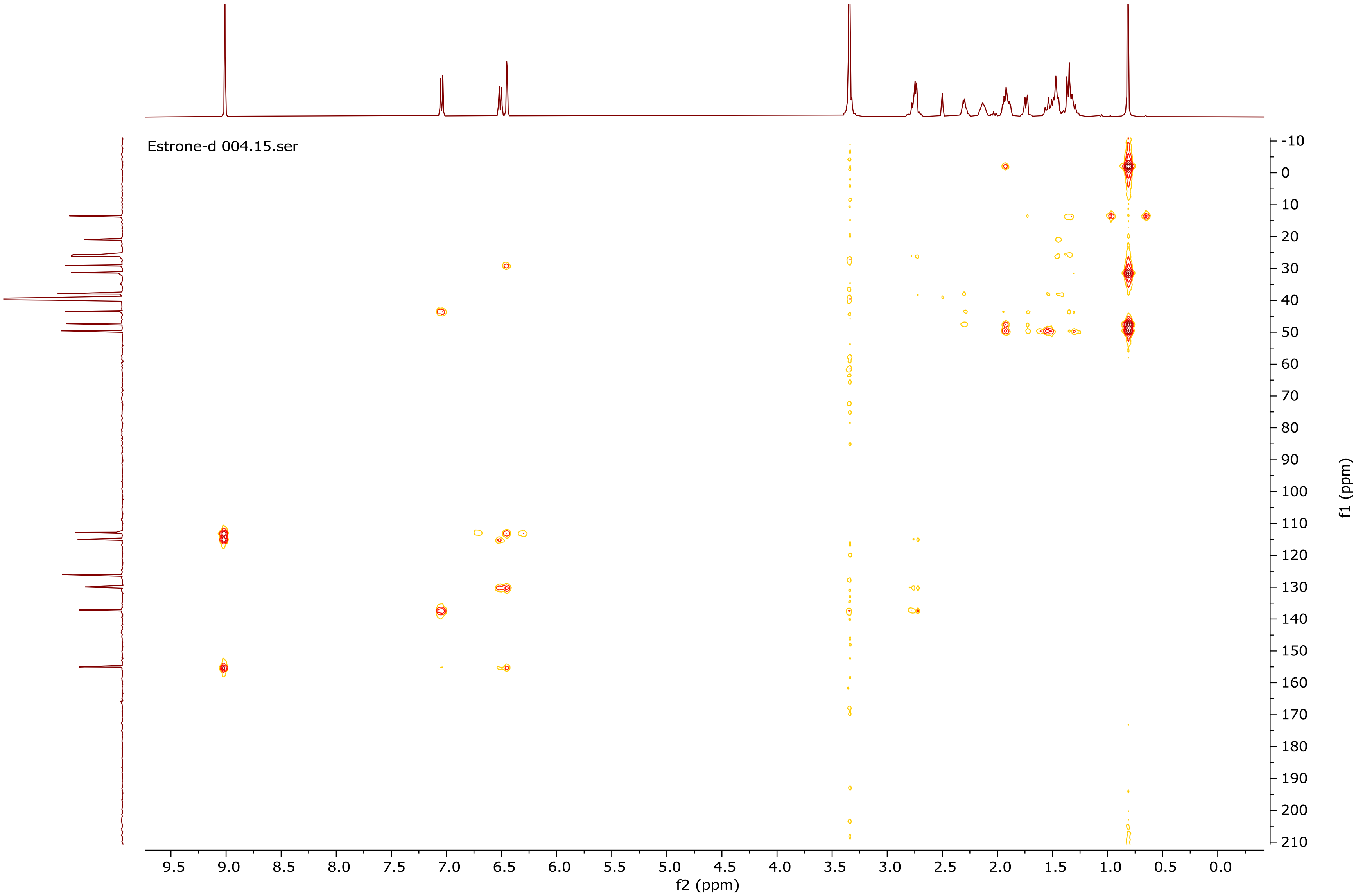

^{1}H NMR spectrum of **20** (400 MHz, DMSO-d$_6$)

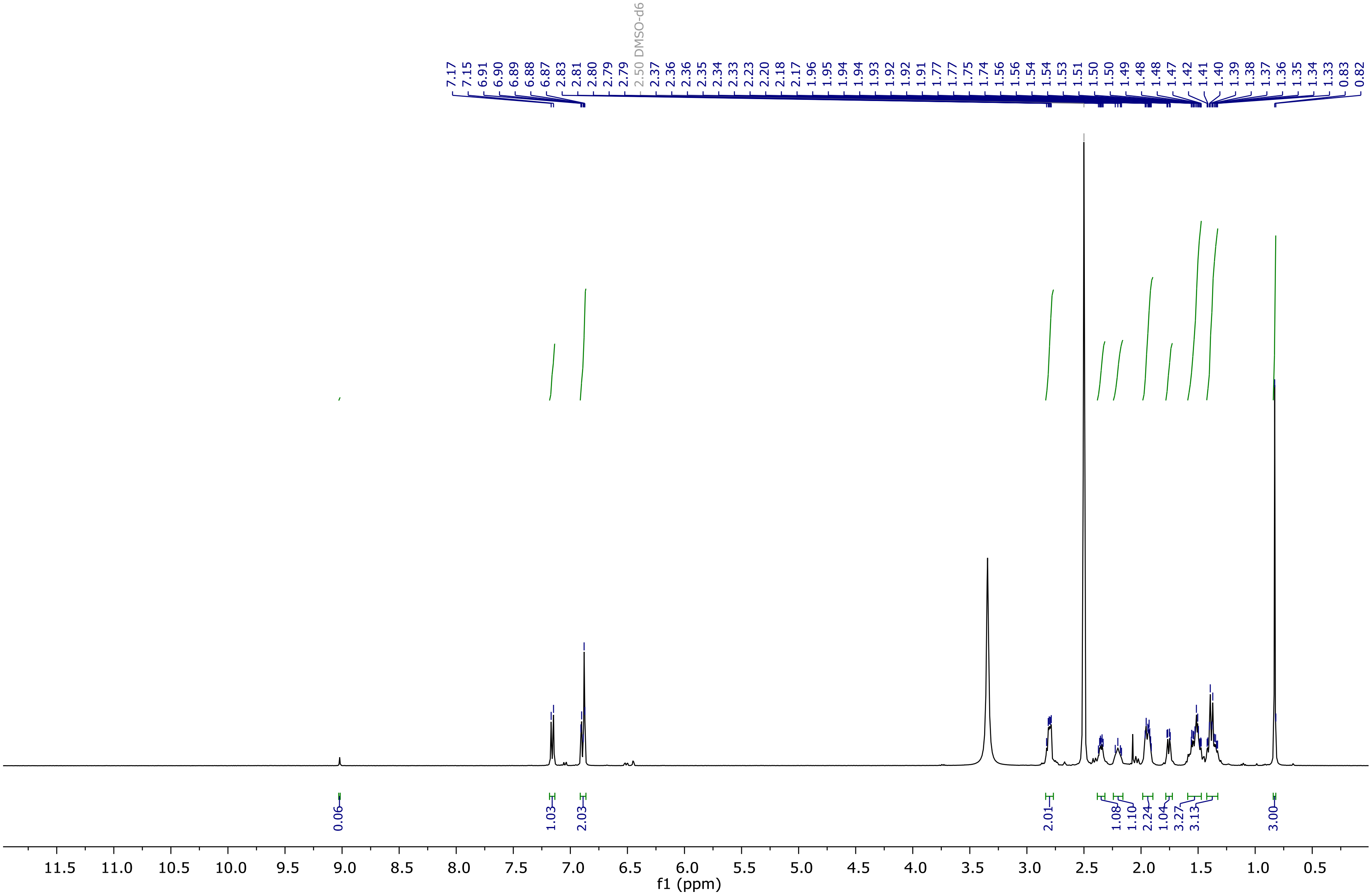

^{13}C NMR spectrum of **20** (101 MHz, DMSO-d_6)

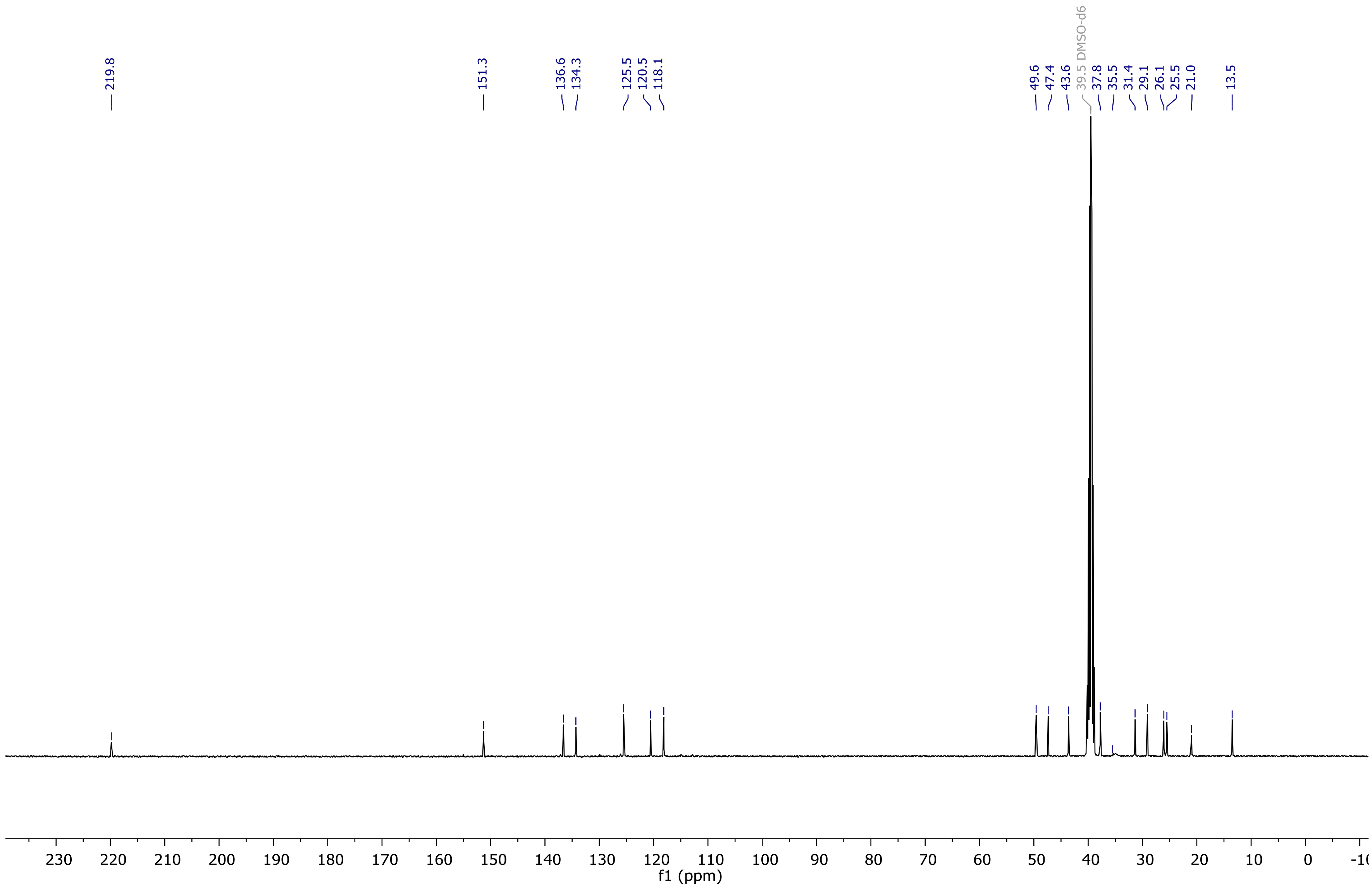

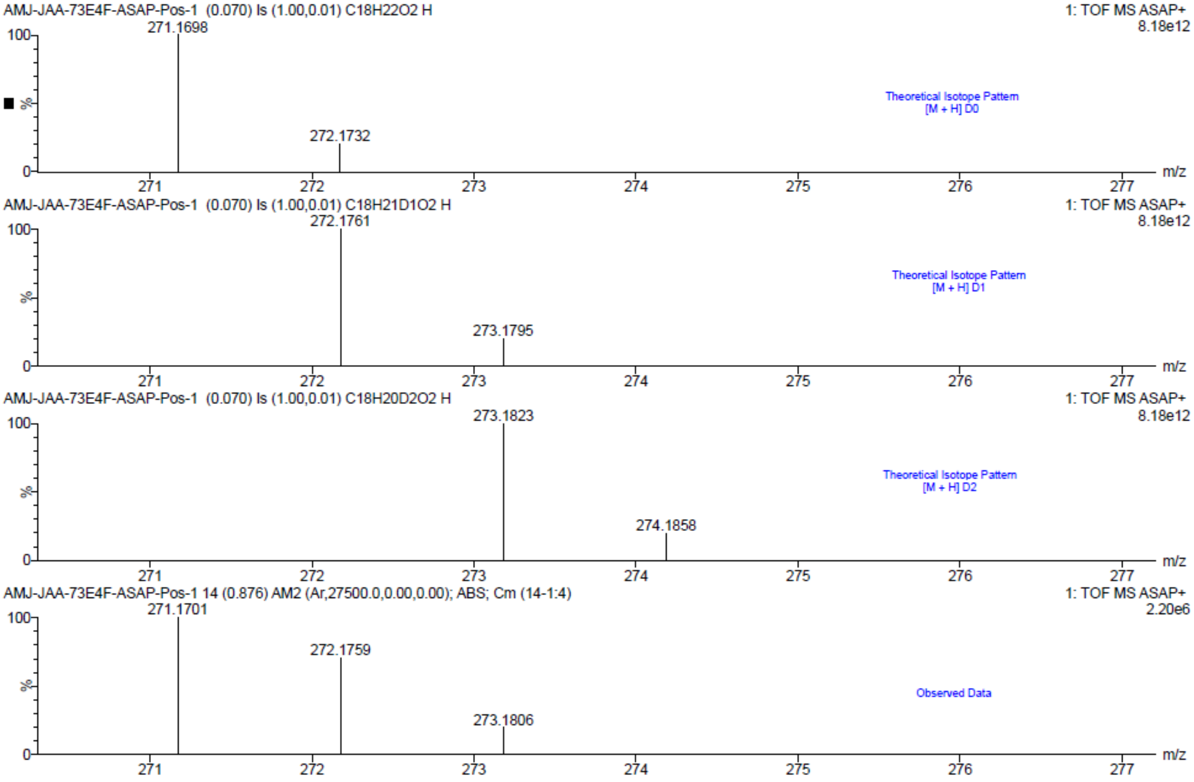

Figure S1. Mass spectroscopic theoretical versus observed deuteration levels for **18**.

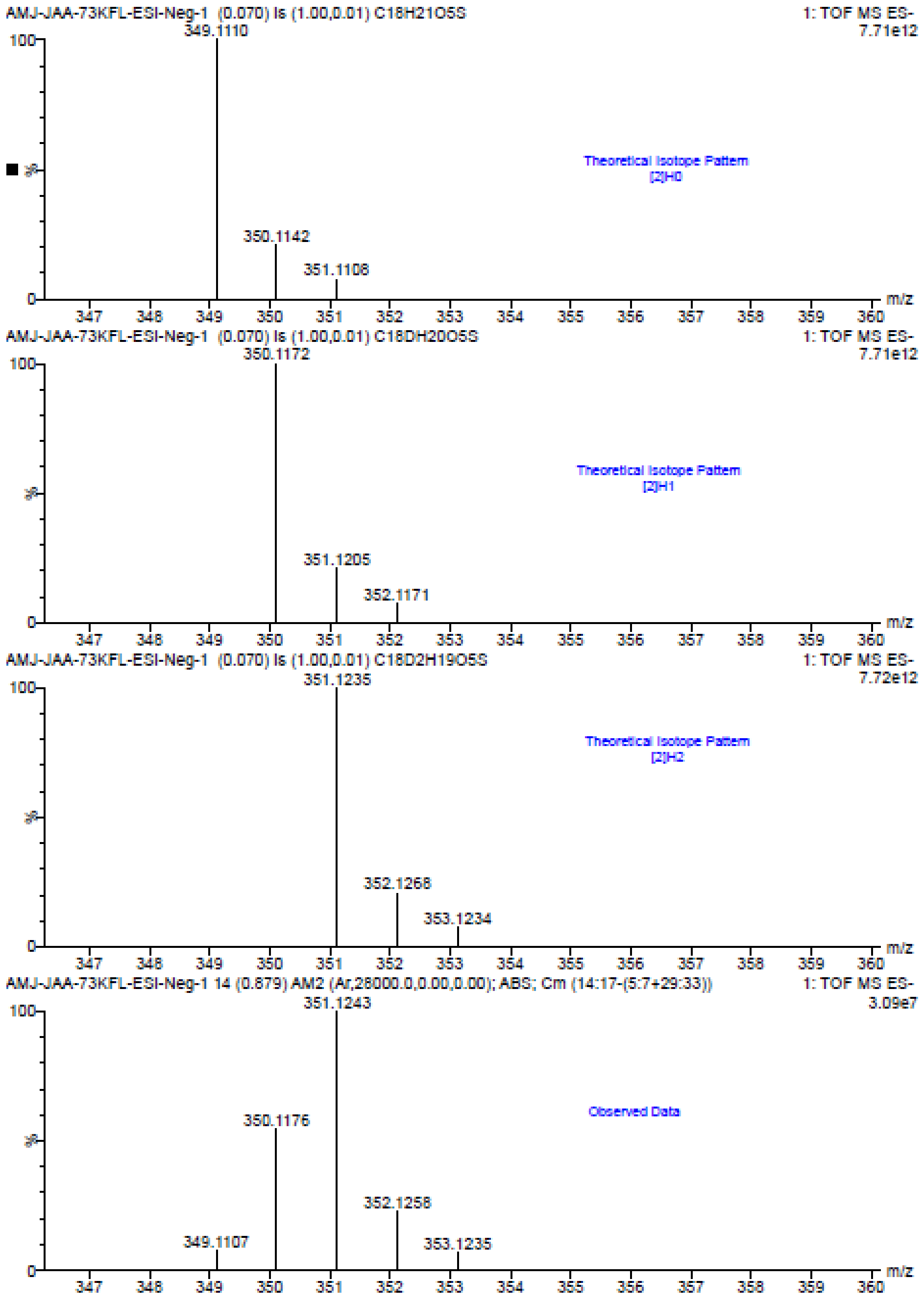

Figure S2. Mass spectroscopic theoretical versus observed deuteration levels for **20**.

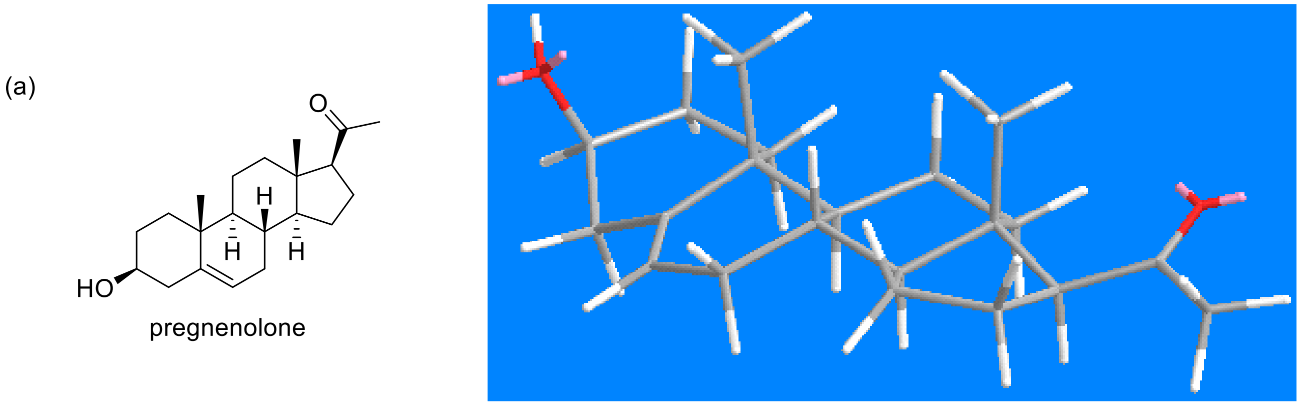

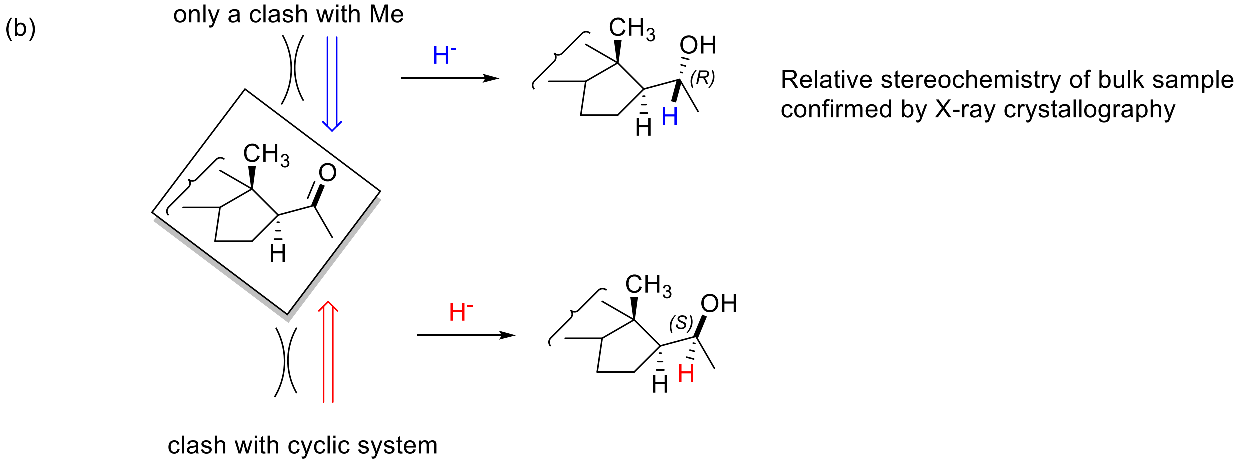

Figure S3. Explanation of observed diastereoselectivity in the ketone reduction of pregnenolone. (a) 3D depiction of pregnenolone and (b) X-ray confirmed outcome is *R*-C(20)-OH by relative orientation of stereocentres This can be explained by the steric approaches of borohydride via the methyl group on the top face or the steroidal framework from the lower face.

X-ray crystallographic data for compound **9**

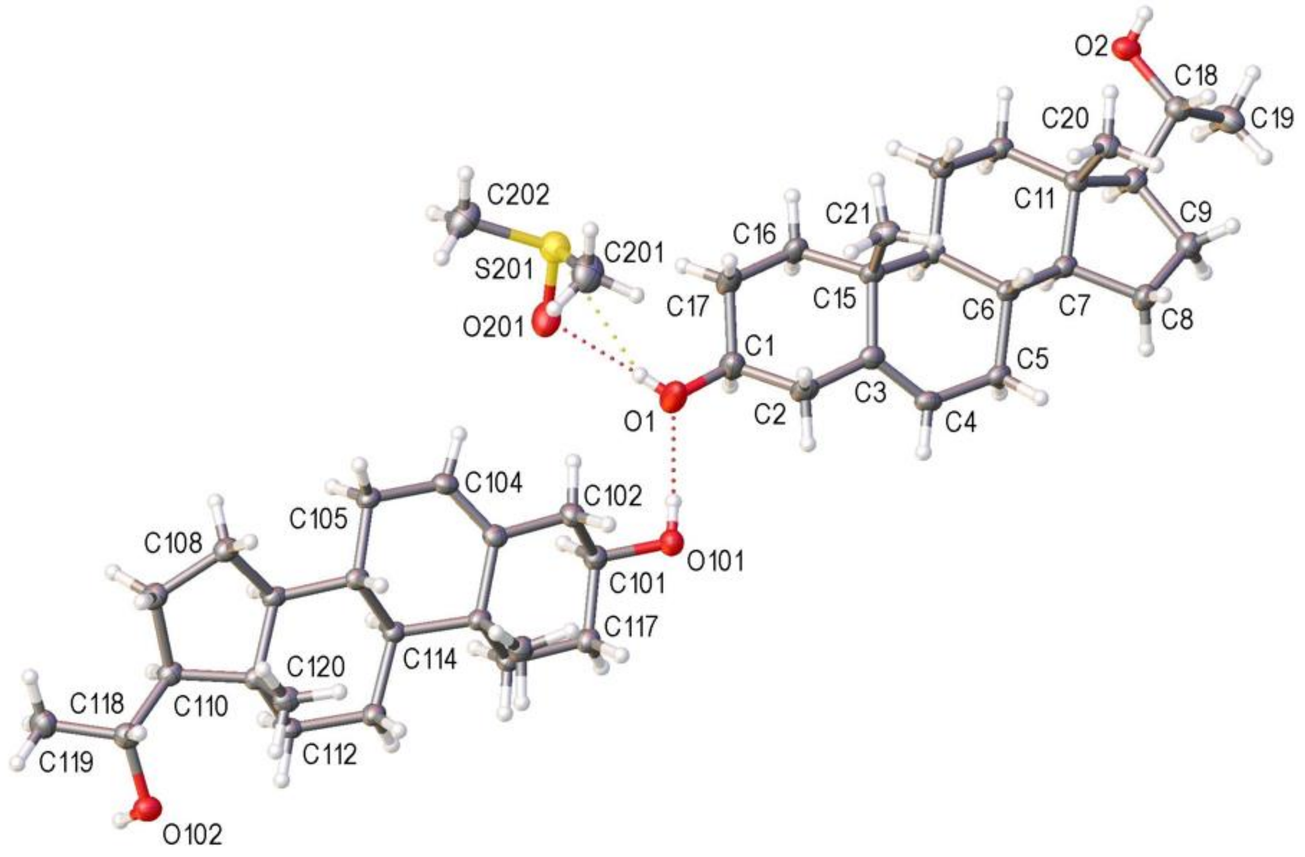

Figure S4 Crystal structure of compound **9** with ellipsoids drawn at the 50 % probability level. Hydrogen bonding within the asymmetric unit is shown using dotted lines.

Table S1 Crystal data and structure refinement for 9.

Identification code	9
Empirical formula	$C_{22}H_{37}O_{2.5}S_{0.5}$
Formula weight	357.54
Temperature/K	99.99(10)
Crystal system	monoclinic
Space group	$P2_1$
a/Å	9.70930(10)
b/Å	14.5298(2)
c/Å	14.3683(2)
α/°	90
β/°	97.7380(10)
γ/°	90
Volume/Å^3	2008.54(4)
Z	4
ρ_{calc}g/cm^3	1.182

μ/mm^{-1}	1.045
F(000)	788.0
Crystal size/mm^3	0.262 × 0.044 × 0.015
Radiation	Cu Kα (λ = 1.54184)
2Θ range for data collection/°	6.208 to 155.712
Index ranges	$-11 \leq h \leq 12$, $-17 \leq k \leq 17$, $-17 \leq l \leq 18$
Reflections collected	44998
Independent reflections	7805 [R_{int} = 0.0568, R_{sigma} = 0.0342]
Data/restraints/parameters	7805/1/475
Goodness-of-fit on F^2	1.051
Final R indexes [I>=2σ (I)]	R_1 = 0.0369, wR_2 = 0.0982
Final R indexes [all data]	R_1 = 0.0393, wR_2 = 0.0997
Largest diff. peak/hole / e $Å^{-3}$	0.36/-0.32
Flack parameter	-0.015(9)

Crystal structure determination of **9**: $C_{22}H_{37}O_{2.5}S_{0.5}$ (*M* =357.54 g/mol): monoclinic, space group $P2_1$ (no. 4), *a* = 9.70930(10) Å, *b* = 14.5298(2) Å, *c* = 14.3683(2) Å, *β* = 97.7380(10)°, *V* = 2008.54(4) $Å^3$, *Z* = 4, *T* = 99.99(10) K, μ(Cu Kα) = 1.045 mm^{-1}, *Dcalc* = 1.182 g/cm^3, 44998 reflections measured (6.208° ≤ 2Θ ≤ 155.712°), 7805 unique (R_{int} = 0.0568, R_{sigma} = 0.0342) which were used in all calculations. The final R_1 was 0.0369 (I > 2σ(I)) and wR_2 was 0.0997 (all data). Flack: -0.015 (9).

The structure contains two crystallographically independent molecules and a molecule of DMSO. The structure occupies a chiral space group and has been confirmed from the diffraction data as being enantiometricaly pure, with a Flack parameter of -0.015(9).

Thus C(1), C(6), C(7), C(10), C(11), C(14), C(101), C(106), C(107), C(110), C(111), C(114) are confirmed as *S* while C(15), C(18), C(115) and C(118) are *R*.

A suitable crystal was selected and a dataset for 9 **was** measured on an **XtaLAB Synergy, Dualflex** diffractometer using a HiPix detector. The data collection was driven and processed and an absorption correction was applied using CrysAlisPro.[S9] The structure was solved using ShelXT [S10] and was refined by a full-matrix least-squares procedure on F^2 in ShelXL.[S11] Figures and reports were produced using OLEX2.[S12] All non-hydrogen atoms were refined with anisotropic displacement parameters. The hydrogen atoms bonded to O(1), O(2), O(101) and O(102) were located in the electron density and the positions refined. All remaining hydrogen atoms were fixed as riding models and the isotropic thermal parameters (U_{iso}) of all hydrogen atoms were based on the U_{eq} of the parent atom.

CCDC 2120263 contains the supplementary crystallographic data for this paper. These data can be obtained free of charge from The Cambridge Crystallographic Data Centre via www.ccdc.cam.ac.uk/data_request/cif.

References

S1. R. Roskoski, *Biochem. Mol. Biol. Educ.* 2002, **30**, 338–339.

S2. D. M. Gill, L. Male and A. M. Jones, *Chem. Commun*. 2019, **55**, 4319–4322.

S3. S. Maeda. *Jpn. Kokai Tokkyo Koho.* 1975, JP 50024261 A 19750315.

S4. X. Wang, H. Liu, P. Yan, J. Liu, Y. Li, Q. Sun and C. Wang, *J. Chem. Res*. 2011, **35**, 291–293.

S5. M. Marian, B. Matkovics, Sz. Vargha. *J. Acta Physica et Chemica.* 1971, **17**, 85-89.

S6. V. Foucher, B. Guizzardi, M.B. Groen, M. Light, B. Linclau. *Org. Lett.* 2010, **12**, 680-683.

S7. L. Levin, *J. Biol. Chem.* 1945, **158**, 725–726.

S8. P. S. Kiuru and K. Wähälä, *Steroids*, 2006, **71**, 54–60.

S9. CrysAlisPro, Rigaku Oxford Diffraction, 2015.

S10. G. M. Sheldrick, *Acta Cryst.* 2015, **A71**, 3-8.

S11. G. M. Sheldrick, *Acta Cryst.* 2015, **C71**, 3-8.

S12. O. V. Dolomanov, L. J. Bourhis, R. J. Gildea, J. A. K. Howard and H. Puschmann, *J. Appl. Cryst.* 2009, **42**, 339-341.
